# Broadband impedance match to two-dimensional materials in the terahertz domain

**DOI:** 10.1038/s41467-017-02336-z

**Published:** 2017-12-20

**Authors:** Phi H. Q. Pham, Weidong Zhang, Nhi V. Quach, Jinfeng Li, Weiwei Zhou, Dominic Scarmardo, Elliott R. Brown, Peter J. Burke

**Affiliations:** 1 Department of Electrical Engineering and Computer Science, University of California, Irvine, CA 92697 USA; 2 Department of Physics and Electrical Engineering, Wright State University, Dayton, OH 45435 USA

## Abstract

The coupling of an electromagnetic plane wave to a thin conductor depends on the sheet conductance of the material: a poor conductor interacts weakly with the incoming light, allowing the majority of the radiation to pass; a good conductor also does not absorb, reflecting the wave almost entirely. For suspended films, the transition from transmitter to reflector occurs when the sheet resistance is approximately the characteristic impedance of free space (*Z*_0_ = 377 Ω). Near this point, the interaction is maximized, and the conductor absorbs strongly. Here we show that monolayer graphene, a tunable conductor, can be electrically modified to reach this transition, thereby achieving the maximum absorptive coupling across a broad range of frequencies in terahertz (THz) band. This property to be transparent or absorbing of an electromagnetic wave based on tunable electronic properties (rather than geometric structure) is expected to have numerous applications in mm wave and THz components and systems.

## Abstract (web summary)

Efficient coupling of an electromagnetic wave to a thin conductor relies on the sheet conductance of the given material. Here, the authors demonstrate that engineering the monolayer graphene sheet resistance enables electrical or chemical tuning from the transmission to the absorption regime up to THz frequencies.

## Introduction

Two-dimensional materials provide a class of atomically thin conductors^1–3^ which can be synthesized with area much larger than the electromagnetic (EM) wavelength in the mm wave and THz bands, creating opportunities to control EM beams without the need for nanoscale patterning that is typically required in the optical^4, 5^. To date, unpatterned monolayer graphene devices have operated exclusively in the weakly coupled, highly transmissive regime^6–18^.

Here, by deliberate engineering of the sheet conductance using large-domain graphene films in combination with chemically modified substrates (to decrease interface scattering), as well as chemical and electrical doping, we fabricate monolayer graphene devices with sheet resistance crossing the characteristic impedance of free space, 377 Ω/□ Achieving this, we show that monolayer graphene can be tuned to behave as a strong absorber over a broadband frequency range, approaching the theoretical impedance at which absorption is maximized^19, 20^. In free space, the maximum absorption is 50%, whereas on a substrate it can be greater than 90% depending on the direction of normal incidence, discussed in detail below. Regardless of the case (free space or on dielectric), the absorption is much larger than the 2.3% optical value^21^, and thus represents a milestone of coupling electromagnetic waves to an atomically thin nanomaterial. This is measured over an extremely broad range, from mm wave to THz frequencies. Strong EM absorption using a single atomic layer exemplifies the fundamental relationship between nanoscale electronics and classical electromagnetism.

## Results

### THz conductance

**Fig. 1:**
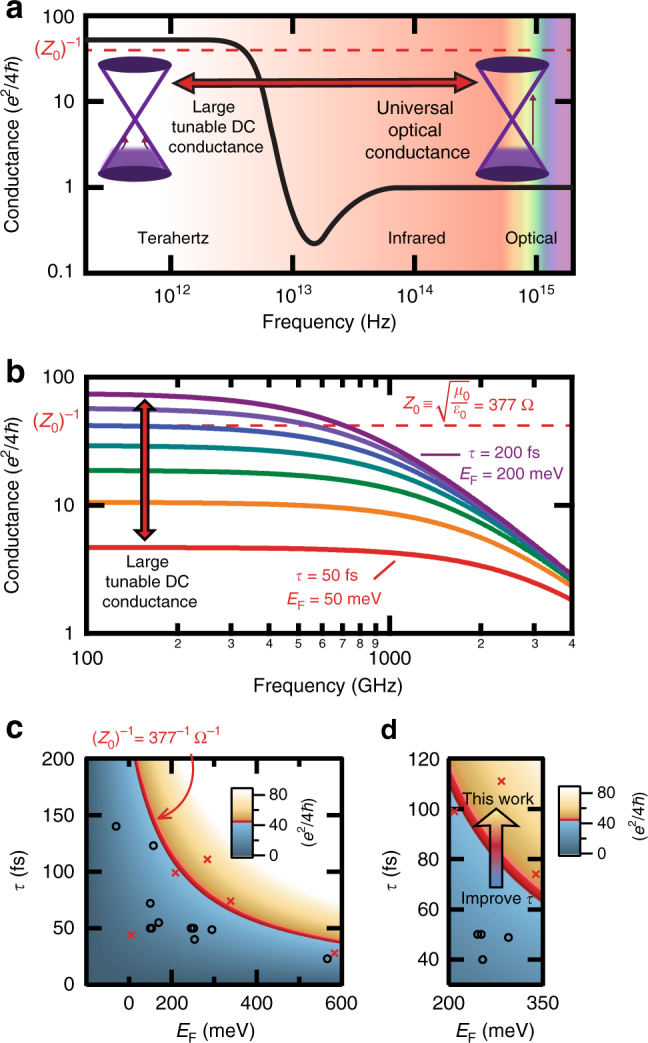
THz conductance. **a** Qualitative trend of frequency-dependent AC conductance for monolayer graphene with normalization to *e* ^2^/4*ħ*. The optical frequency range exhibits a universal value of 1, while in the THz range, the AC conductance can be orders of magnitude higher. **b** Theoretical trend of THz AC conductance for monolayer graphene plotted with a linear variation of the *E*_F_, and *τ*. Graphene samples can cross the free space conductance value, (*Z*_0_)^−1^, within the THz regime. **c** Device design plot showing the graphene sheet conductance (at 100 GHz) in units of (*e* ^2^/4*ħ*) for changes in *E*_F_ and *τ*. The (*Z*_0_)^−1^ threshold is set as red on the color scale. Previous graphene THz device parameters are plotted as circles, whereas those from this work are denoted as *x*’s. **d** By decreasing the electron scattering in our devices to increase *τ*, the (*Z*_0_)^−1^ threshold can be crossed into the absorption-dominating regime

The zero-gap band structure of graphene leads to a non-trivial frequency-dependent AC conductance, which governs the absorption of the EM radiation. As depicted in Fig. 1a, at optical frequencies the sheet conductance of graphene is dominated by interband transitions, and exhibits a universal value of *G*_g_ = (*π/*4)·*G*_0_ = (*e* ^2^/4*ħ*)^21^, where *G*_0_ is the conductance quantum. This results in its low absorbance, approximated by the product of *G*_g_ and the characteristic impedance of free-space, *Z*_0_ = 377 Ω. This product can be written *π*·(*e* ^2^/*ħc*) ≡ *π·α*, where *α* is the fine structure = 1/137. Thus, only ≈ 2.3% of the light is absorbed, and the majority of the light is transmitted.

In contrast, at frequencies lower than ~ 2*E*_F_/*h* (where *E*_F_ is the Fermi energy), as is the case in the THz range, the photon energy is too low to excite electron–hole pairs, and the electromagnetic sheet conductance is expected to follow the DC sheet conductance^22, 23^. This should occur for frequencies up to ~ 1/*τ*, where *τ* is the scattering time, at which point the conductance is expected to undergo a Drude-like roll-off^6–9^, dropping towards the low optical value, (Fig. 1a). The AC conductance can be adjusted by engineering *E*_F_ and *τ* according to the equations:

1 $$G_{{\mathrm{DC}}} = \frac{{e^2}}{{4\hbar }}\frac{4}{\pi }\frac{{E_{\mathrm{F}}\tau }}{\hbar }$$

2 $$G\left( \omega \right) = \frac{{G_{{\mathrm{DC}}}}}{{1 + i\omega \tau }}$$

This creates the opportunity to adjust the THz conductance into the regime where the majority of the incident light is absorbed (*A* > *R*, *T*). This prospect is indicated schematically within the THz frequency range in Fig. 1b, where hypothetical conductance curves for plausible *E*_F_ and *τ* values are plotted. Figure 1c presents “device design” charts (calculated for 100 GHz), which show how combinations of *E*_F_ and *τ* affect the AC conductance; the absorption-dominating regime begins when the sheet resistance ≤ 377 Ω/□ (set as red on the *z*-axis color scale). Previous investigations of single-layer graphene THz absorption fail to surpass the *Z*_0_ = 377 Ω threshold^6–18^ (denoted as circles in Fig. 1c), even in the case where *E*_F_ was designed to be purposefully large (using heavy chemical doping^6, 15^, or strong electrical gating^6, 7, 10, 12, 13, 15^). Instead, as demonstrated in this work and emphasized as an arrow in Fig. 1d, by carefully fabricating devices with improved *τ*, the graphene conductance can be large (denoted as an ‘*x*’ in Fig. 1c), and the device sheet resistance can be tuned to be below the free space impedance threshold. We successfully surpass this threshold with a graphene sheet, and reach near maximum absorption.

### Transmission, reflection and absorption regimes

**Fig. 2:**
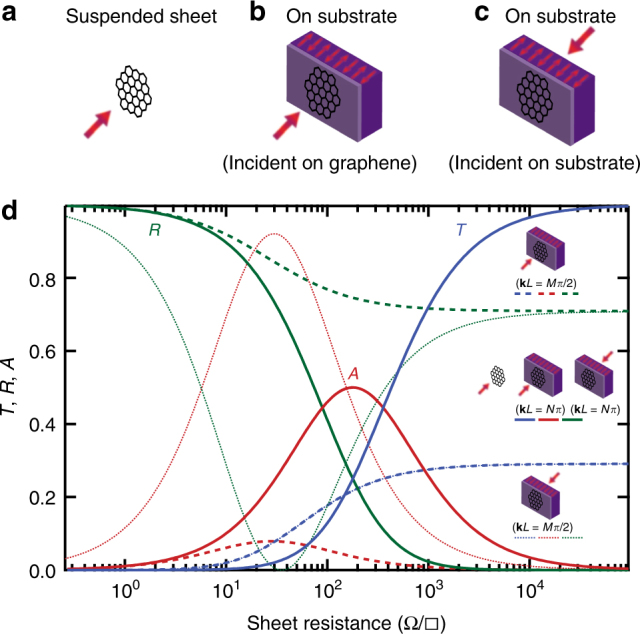
Transmission, reflection and absorption. **a**–**c** A suspended film and a film on a substrate for two incidence directions were analyzed using the transmission line model. *n* = 3.41 was used as the substrate index of refraction for calculation. **d** The suspended film/Woltersdorff equations (plotted as solid lines) for *T*, *R* and *A* vs. sheet resistance were compared to a film on a substrate. When the half-wave resonance condition is satisfied (**k** *L* = *Nπ*, where *N* is an integer), the equations for *T*,* R* and *A* reduce to exactly the Woltersdorff equations (plotted for both directions as solid lines, and are indistinguishable from the suspended film). Hence, analyzing the device at half-wave resonance provides a direct comparison of the graphene absorption on a substrate to that of a suspended film. At quarter-wave resonance (**k** *L* = *Mπ*/2, where *M* is an odd integer), *T* is the same for both incidence directions, but *R* and *A* are different for each incidence direction (plotted as the dotted lines)

Our measurement technique consists of using an ultra-broadband THz spectrometer, based on the photomixing of two optical lasers to generate a coherent THz beam^24^. This system (block diagram shown in Supplementary Fig. 1) enables continuous transmission measurement from 200 GHz to 1.2 THz with over 60 dB dynamic range at the low end, and up to 40 dB at the high end. The graphene films are mounted on high-resistivity silicon substrates of thickness ~ 400 μm, which do not absorb significantly in the THz region. Because of the difference in the index of refraction between the silicon substrate (*n* ≈ 3.4) and air (*n* = 1), there is an etalon effect present in the frequency-dependent transmission (even in the absence of graphene). With graphene present, we can model the effect of varying the graphene sheet resistance to calculate the net transmittance using the transmission line model (Supplementary Note 1–4), and compare these values (for a film on a substrate with incidence in both directions) to that of a suspended graphene film (Fig. 2a–c).

A plane wave can be transmitted (*T*), reflected (*R*) or absorbed (*A*). Electromagnetics states that a perfect conductor is a mirror (*T* = 0; *R* = 1;* A* = 0) and a perfect insulator is a transmitter (*T *≤ 1, *R* ≥ 0, *A* = 0). What is the quantitative relationship between* T*, *R*, *A* and the sheet resistance? The three are not completely independent, as *T* + *R* + *A* must equal unity due to conservation of energy. The relationship for the transmittance, reflectance, and absorptance of a suspended thin metallic film was derived in 1934^19^, and is given by,

3 $$T = \frac{{4g^2}}{{(1 + 2g)^2}}$$

4 $$R = \frac{1}{{(1 + 2g)^2}}$$

5 $$A = \frac{{4\,g}}{{(1 + 2g)^2}}$$

6 $$g \equiv {\raise0.5ex\hbox{$\scriptstyle {R_\square }$}\kern-0.1em/\kern-0.15em \lower0.25ex\hbox{$\scriptstyle {Z_0}$}}$$

These expressions show the change from transmission-dominating to absorption-dominating occurring at the critical threshold of *Z*_0_ = 377 Ω. Within the absorption-dominating regime, the peak absorption occurs when the sheet resistance is equal to *Z*_0_/2 (Fig. 2d).

When mounted on a substrate of index of refraction, *n*, numerous considerations such as direction of normal incidence (on the graphene or substrate side first) and substrate thickness govern *T*, *R* and *A*. Furthermore, interference effects from the substrate cause Fabry–Perot behavior depending on the THz frequency (*f*), substrate thickness (*L*) and index of refraction (*n*). When incident on the graphene side first, the general analytical expressions for *T*, *R* and *A* are given by,

7 $$T = 4Z_0\left| {\frac{{gn\left( {n\,{\mathrm{sin}}\left( {{\mathbf{k}}L} \right) - i\,{\mathrm{cos}}\left( {{\mathbf{k}}L} \right)} \right)}}{{\left( {1 + g + gn^2} \right)Z_0\,{\mathrm{sin}}\left( {{\mathbf{k}}L} \right) - i\left( {1 + 2g} \right)nZ_0\,{\mathrm{cos}}\left( {{\mathbf{k}}L} \right)}}} \right|^2 \\ \frac{{2Z_0}}{{1 + n^2 - {\mathrm{cos}}\left( {2{\mathbf{k}}L} \right)\left( {n^2 - 1} \right)}}$$

8 $$R = \left| {\frac{{\left( {1 - g + gn^2} \right){\mathrm{sin}}\left( {{\mathbf{k}}L} \right) - in{\mathrm{cos}}({\mathbf{k}}L)}}{{\left( {1 + g + gn^2} \right){\mathrm{sin}}\left( {{\mathbf{k}}L} \right) - i\left( {1 + 2g} \right)n\,{\mathrm{cos}}({\mathbf{k}}L)}}} \right|^2$$

9 $$A = 4gZ_0^2\left| {\frac{{{\mathrm{sin}}\left( {{\mathbf{k}}L} \right) - in\,{\mathrm{cos}}({\mathbf{k}}L)}}{{\left( {1 + g + gn^2} \right)Z_0\,{\mathrm{sin}}\left( {{\mathbf{k}}L} \right) - i\left( {1 + 2g} \right)nZ_0\,{\mathrm{cos}}({\mathbf{k}}L)}}} \right|^2$$

Similar expressions for incidence on the substrate side are given in Supplementary Note 4.

For both incidence directions, when **k** *L* = *Nπ,* where **k**, is the wave vector, *L* is the substrate thickness, and *N* is integer values, or when the THz frequency is at half-wave resonance with the graphene–silicon etalon, which occurs at even integer multiples of $$f = \frac{c}{{\left( {4*n*L} \right)}}$$, the equations for *T*, *R* and *A* reduce to exactly the same as the Woltersdorff equations^19^ (Eqs. 3–5). Hence, for a broadband transmittance measurement, at half-wave resonance, or at the transmittance maxima (up to a critical sheet resistance value, after which the half-wave resonance occurs at the transmittance minima, see Supplementary Note 3 and Supplementary Fig. 4e), the behavior of the graphene film can be directly compared to a suspended film, where the onset of strong absorption occurs at *Z*_0 _= 377 Ω, with maximum absorption of 50% occurring at *Z*_0_/2. In Fig. 2d the half-wave resonance condition for *T*, *R* and *A* are indistinguishable from those of the suspended case.

In contrast, at quarter-wave resonance, when **k** *L = Mπ*/2, where *M* is odd integer values, the sheet resistance-dependent transmission through the graphene–silicon etalon remains low (transmission never dominates). Although the transmittance is low and equivalent for both incidence directions (graphene or substrate first), the sheet resistance-dependent reflection and absorption indeed depend on the incidence direction. When the THz beam is incident on the graphene side first, interference effects cause the device to behave as mostly reflecting (always reflection dominating), resulting in low absorption. On the other hand, when the THz beam is incident on the substrate first (with graphene on the backside), the absorption, transmission and reflection can be affectedly tuned by the sheet resistance. This allows the absorption maximum to increase to > 50% for low sheet resistance values (Supplementary Fig. 5a).

The two extreme cases (half-wave resonance and quarter-wave resonance) shows contrasting behavior, but like the suspended film, there still exists certain impedance values that determine the peak absorption, and the onset of the absorption-dominating threshold (Fig. 2d). The absorption-dominating threshold and peak absorption can shift (to even lower sheet resistance values) depending on the substrate index of refraction. We have calculated and plotted this trend for various values of the index of refraction in Supplementary Fig. 5b. Regardless of whether the graphene film is suspended or on a substrate, the largest sheet resistance value (most resistive) in which the film becomes absorption dominating is 377 Ω/□, and hence this value signifies the doorway to strong absorption. Detailed below, we observe a decrease in the broadband transmittance as the graphene sheet resistance is tuned below 377 Ω/□, signifying absorption approaching the 50% maximum for a THz beam incident on a graphene sheet on substrate.

### Graphene device fabrication on modified substrate

**Fig. 3:**
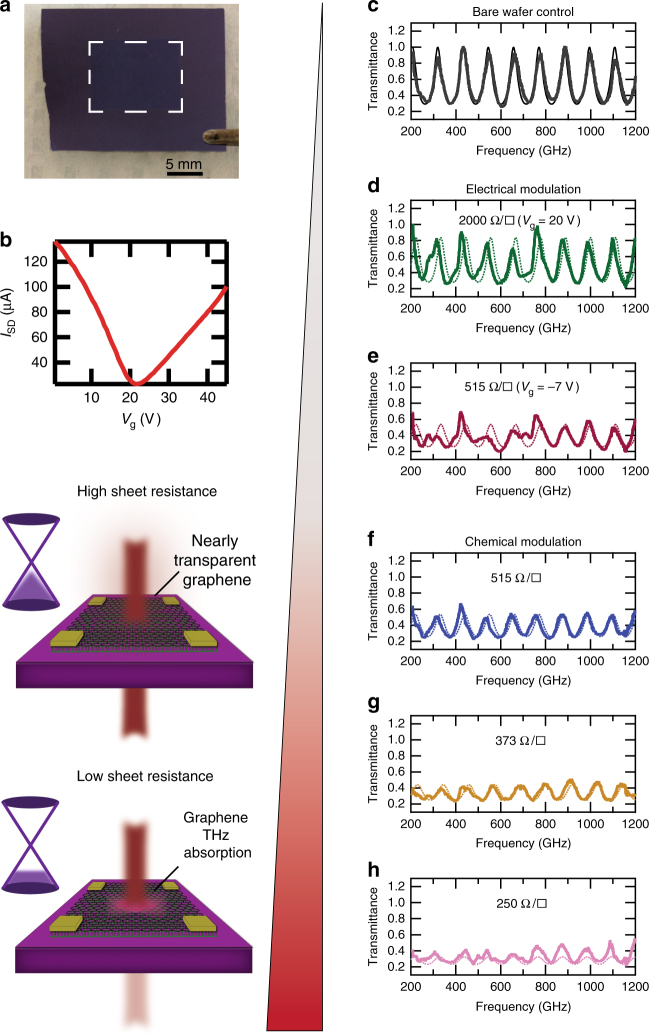
Broadband impedance matching. **a** Optical photograph of graphene transferred onto ODTS modified oxide on high-resistivity silicon substrate. The scale bar is ~ 5 mm. **b** Source-drain current vs. gate voltage for typical graphene on ODTS SAM device. The charge neutral point is ~ 22 V. **c** Transmittance vs. frequency data acquired for a control sample (bare high-resistivity silicon substrate with no graphene). **d**, **e** Transmittance vs. frequency data for a graphene sample gated at 20 V and −7 V. **f**–**h** Transmittance vs. frequency for three devices after chemical doping to modify the zero-bias sheet resistance. The expected transmittance values assuming the DC conductance as the AC contribution (assuming negligible susceptance) are plotted as dotted lines

Previously, researchers used bare, unpatterned graphene, and measured the THz resistance to be greater than 377 Ω^6–18^. This includes our previous work^9^, where the graphene–etalon structures were fabricated using chemical vapor-deposited graphene films^25^ transferred^26^ onto a 90 nm gate oxide layer on high-resistivity silicon substrates. To improve *τ*, so as to increase the conductance of the devices, we synthesized large-domain (mm-sized) graphene films (to minimize graphene grain boundaries)^27^, in addition to depositing octadecyltrichlorosilane (ODTS) self-assembled monolayers (SAM) on the wafer (to decrease scattering)^28^ prior to graphene transfer (Supplementary Note 5). Across the measured frequency range, the SAM does not significantly affect the THz absorption (Supplementary Note 6). The resulting air/graphene/substrate structure shown in Fig. 3a was used for THz transmission measurements with the THz beam incident on the graphene side first. When gating the graphene to the charge neutral point (CNP) without the use of a SAM, large voltages (> 50 V) must be applied to the high-resistivity silicon layer. Utilization of the SAM layer provides significant improvement of the transconductance of the graphene film, and a smaller operating voltage window (Supplementary Fig. 7b–c). The depletion curve shown in Fig. 3b shows a CNP at ~ 22 V, and indicates slight *p*-doping of the graphene film. We performed a series of THz transmission measurements on these devices for a variety of sheet resistance values, as shown in Fig. 3d–h.

### Electrical modulation of broadband THz

In order to de-convolve the etalon effect, the broadband transmission through a bare high-resistivity silicon substrate (without graphene) is first measured as a control. The multiple interference peaks of transmission around ≈ 1.0 (Fig. 3c) confirm the low-loss nature of the bare substrate. Upon the addition of a monolayer sheet of graphene on the ODTS SAM buffer layer (and when applying a voltage on the silicon substrate near the *V*_CNP_ (≈ 20 V) as shown in Fig. 3d), the graphene film behaves as a nearly transparent film (sheet resistance ≈ 2 kΩ/□), as the amplitudes of the transmission peaks approach unity. The theoretical graphene-on-Si etalon transmittance vs frequency curves, calculated for an AC admittance that is purely real (susceptance equal to zero) and equal to the measured DC conductance, are plotted as dotted lines for comparison, and generally agree with the measured values. This is clearly in the transmission-dominating regime, as the presence of the graphene has little effect on the THz transmission through the sample.

We next demonstrate the ability to electrically tune to the absorption-dominating regime. By applying −7 V, the graphene DC sheet resistance is decreased to ≈ 515 Ω/□, and we observe considerably decreased maxima in the transmission peaks (Fig. 3e). Despite the fact that the gate voltage drop across the graphene film is small compared to the high-resistivity silicon substrate, consistent with the *I*–*V* curve (Fig. 3b), our device still enables significant control of the single-layer graphene sheet resistance, which results in a large transmittance variation of the incident THz radiation. The gate-modified transmittance peak values are plotted together with the *I*–*V* characteristics in Supplementary Fig. 8a for an additional device. Gate leakage through the oxide and ODTS SAM ([10^−5^] A) was observed at voltages beyond this range, and hence gate voltages were restricted to −7 V to +45 V. Across the broadband frequency range measured, the transmittance variation at the peaks can be used to define a depth of modulation (DoM) (relevant to the use as a spatial-light modulator), defined as (*T*_High_ *−T*_Low_)/*T*_High_. The DoM is substantial, with a maximum value of ≈ 52% at the ~ 330 GHz peak (Supplementary Fig. 8b). For a simple, unpatterned device geometry, consisting of only one monolayer, this is the largest transmittance variation that has been reported to date for a graphene-based modulator, to the best of our knowledge^9, 10, 12, 15, 17^. A 52% DoM is comparable to other patterned graphene devices such as extraordinary optical transmission structures^29^ and periodic arrays of graphene nanodisks^30^. Compared to patterned device geometries, which usually create frequency- or polarization-dependent behavior, our device structure (which does not require any lithography) allows for useful THz modulation over 1 THz of frequency range with polarization independence. This result was independently confirmed in two different labs (Supplementary Note 7).

### Impedance matching using chemical doping

**Fig. 4:**
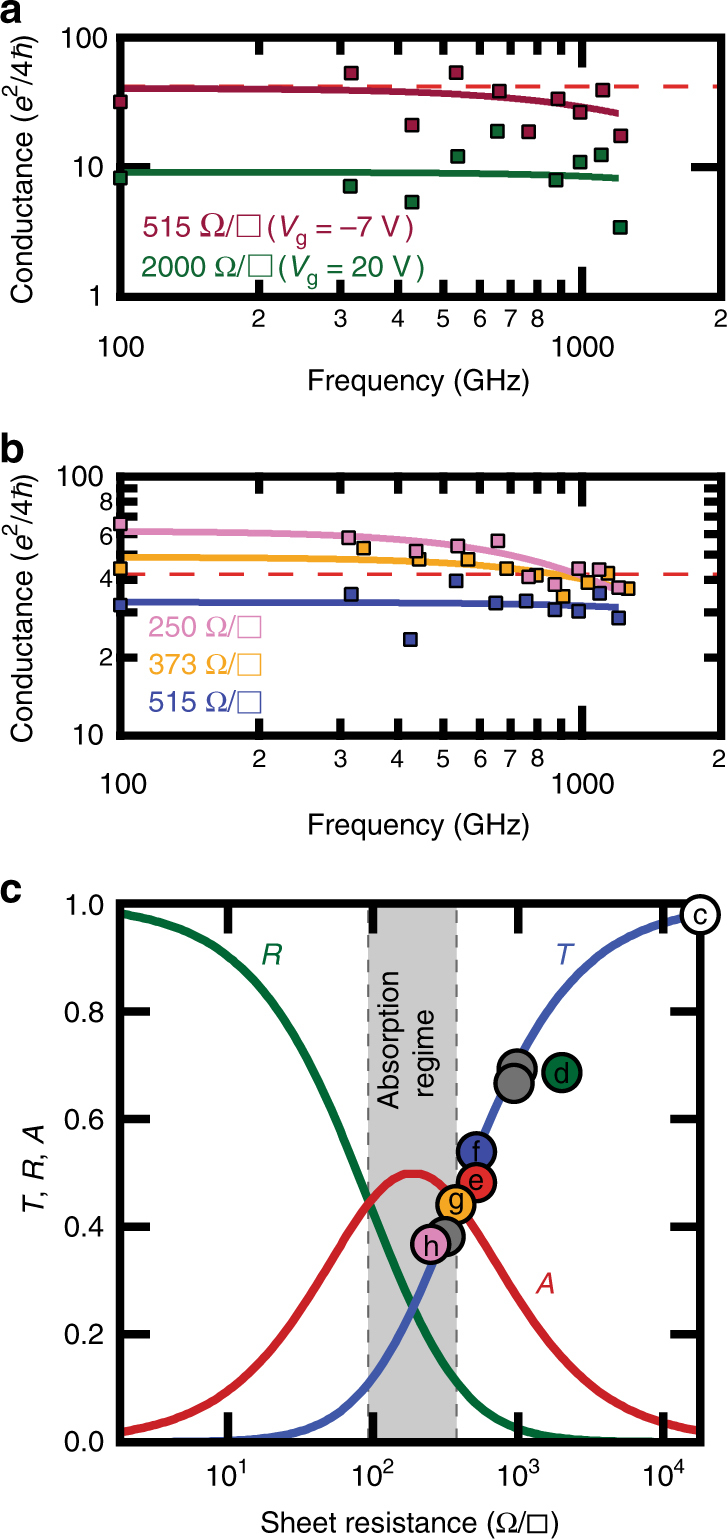
Drude conductance and strong absorption. **a**, **b** The AC conductance values calculated by fitting the measured transmittance peaks for both electrically and chemically modified devices from Fig. 3 are plotted as squares. Drude model trends estimated from the frequency-dependent AC conductance data are plotted as solid lines. **c** The measured transmittance peak values (at ~ 655 GHz) are plotted as a function of sheet resistance. Colored (labeled) circles represent data from devices shown above, gray circles are from devices measured, but not displayed in Fig. 3, and the white circle represents the control sample. The theoretical transmittance, absorbance and reflectance values calculated from the half-wave resonance case for a device on a substrate are plotted as solid lines. The transmittance value becomes less than the absorbance value at sheet resistance values < 377 Ω/□, marking the beginning of the absorption regime

Figure 4a shows the inferred frequency-dependent conductance varying from much less than (*Z*_0_)^−1^ (green curve, *V*_g_ = +20 V, 10× less than (*Z*_0_)^−1^), to approximately equal to (*Z*_0_)^−1^ (red curve, *V*_g_ = −7 V), and clearly demonstrates electrical tuning from the transmission to absorption-dominating regimes. In order to achieve the necessary sheet conductance values near maximum absorption, and beyond (*Z*_0_)^−1^, we utilized chemical (rather than electrical) tuning. We intentionally dope graphene films to have low sheet resistance in the zero-bias state (to mitigate inconsistencies that may arise from the inhomogeneous gating using a high-resistivity substrate, and to ensure no gate leakage occurs) by using benzimidazole (BI) dissolved into the copper etching solution during graphene transfer^31^ (Supplementary Note 8). Figure 3f–h shows the broadband transmittance vs. frequency for three graphene films having zero-bias DC sheet resistances of 515, 373 and 250 Ω/□, respectively. Similar to the electrical modulation, we observe a steady decrease in the THz transmittance peaks. The lowest sheet resistance is clearly less than 377 Ω and the trend of reduced transmission with reduced sheet resistance is observed over the entire measurement band.

### Drude conductance and strong absorption

The measured transmission spectra can be used to determine the AC sheet conductance, using the bare silicon control sample as nearly lossless “calibration”^9^ (although it is noted that the transmission peaks do not necessarily remain at a fixed frequency when covered by a thin film). For devices covered by a graphene film, each transmittance peak is fitted to yield a AC admittance (real conductance and imaginary susceptance) value; the measured DC conductance and the estimated THz conductance are then fitted according to Eqs. 1 and 2 to estimate *E*_F_ and *τ* for each device (Supplementary Note 9). The calculated peak conductance values are shown in Fig. 4a, b, with a dotted red line showing the 377 Ω threshold. The predicted Drude conductance using the estimated *E*_F_ and *τ* is plotted as a solid line, and closely matches the measured DC value (shown on the* y*-axis). Supplementary Note 10 summarizes in a graphical representation our achievement crossing the free-space impedance threshold within the mm wave and THz frequency ranges, and compares our data to previous measurements in the literature. In this work, the frequency-domain measurement is especially broad (spanning the sub-THz regime), and the optical conductance and THz absorption of the graphene film are remarkably large for one atomic layer. Supplementary Table 1 shows the extracted Drude parameters, *E*_F_ and *τ*, for measured devices. Notably, the values of *E*_F_ are moderate, while the values of *τ* can be large (> 100 fs), and stress the importance of engineering *τ*, as initially outlined in the device design plots in Fig. 1c, d. These figures clearly demonstrate our ability to tune the sheet resistance of monolayer graphene, either electrically or chemically, from well above to below the characteristic impedance of free space. We next focus on our more quantitative demonstration of the relationship between the transmittance and the sheet resistance.

We plot in Fig. 4c the predicted transmission (blue line), reflection (green line) and absorption (red line) for a graphene film as a function of sheet resistance for a film mounted on a silicon substrate, at half-wave resonance satisfying the condition **k** *L* = *Nπ*. At half-wave resonance, the free-space and etalon effect give the same predicted *T*, *R* and *A* vs. sheet resistance (Fig. 2). We also show the measured transmittance peak (centered around ~ 655 GHz) values (color points are from devices measured in Fig. 3, gray points are for devices measured during this work, but not displayed in Fig. 3). The 377 Ω vertical dotted line indicates the characteristic impedance of free-space threshold, and marks the start of the absorption regime, where the fractional absorbance (~ 0.44) of the THz radiation by the graphene film becomes greater than the fractional transmittance (and reflectance)^19^. Here, we have realized the crossover from the transmission regime, and have measured correspondingly small transmittance values for devices within the absorption regime. For our best single-layer device of ≈ 250 Ω/□, the value of the graphene film absorbance is calculated to be ≈ 48.6%: approaching the theoretical limit of 50% (for normal incidence on graphene side first).

## Discussion

Instead of designing the single-layer sheet resistance to cross into the absorption-dominating regime, previous experiments for unpatterned graphene devices boosted low absorbance by the addition of multilayers^32–35^, or by suppressing the transmission channel^36^ using double-pass/reflection/“Salisbury screen” geometries^11, 14^. Other thin film (multilayer) materials have also demonstrated remarkable control of absorption by controlling film thickness, such as niobium nitride (NbN)^37–39^ and indium tin oxide (ITO)^40^. Here, our experiments show that exceptionally large, near maximum, absorption can be reached in the thickness limit of one single layer of graphene.

Impedance matching to the free-space threshold using a double-pass, reflection mode geometry could achieve 100% absorption^34, 41^, and may further enhance the single-layer reflection DoM^11, 42^. Pertaining to designing devices that operate in transmission (such as the work performed here), the suspended film and Woltersdorff equations^19^ stand as the ideal situation for broadband absorption. Using the transmission line model for a film on a substrate, as the substrate index of refraction value approaches *n* = 1, or the substrate thickness approaches, *L* = 0, the amplitudes of the Fabry–Perot fringes decrease, and the widths of the fringes increase (as calculated values converge toward the suspended film/Woltersdorff equations for all frequencies). Although it is shown by calculation^43^ that the (quarter-wave resonance) absorption can be greater than 50% if the THz beam is incident on the substrate side first (Supplementary Note 11), this geometry does not easily allow for use as a transmission-mode modulator, because changes in the sheet resistance have less effect on the transmittance compared to the geometry employed in our experiments. Nevertheless, it could still be useful as a reflection-mode device, but the major challenge of engineering low sheet resistance values remains, since absorption greater than 80% in this geometry requires sheet resistance values of less than 100 Ω/□.

We were able to achieve a large transmission DoM by improving the transconductance (allowing the sheet resistance of a single device to span a broad range) of the graphene on ODTS SAM layer. The high-quality, large-domain graphene films on SAM used here were confirmed via Raman mapping^44, 45^ (Supplementary Note 8), and suggest that substrate modification improves the sheet resistance by improving mobility at high carrier density^28, 46^ (Supplementary Fig. 15). Although other materials can exhibit tunable conductance such as vanadium dioxide (VO_2_)^47–49^, NbN^37–39^, ITO^40^, metal thin films or even multilayer graphite, the conductance of monolayer graphene can be easily tuned via external voltages due to the extremely high mobility. For high-frequency applications, the mobility of single-layer graphene is superior to bilayer (multilayer) graphene^50^, and other semiconducting materials^42, 51^, but fabrication remains a challenge for large-area devices which precludes electronically tunable devices operating at very low sheet resistance (even in the simple case of EM incidence on the substrate side first). A combination of substrate modification to improve mobility with techniques such as electrolyte gating^33^ or device integration^17^ could yield electrically controlled devices with high modulation frequency and low operation voltage (see Supplementary Note 7). Additional improvements in large-area (mm-scale) graphene synthesis and transfer will foster better control from the transmission-to-absorption regimes (towards the reflection-dominating regime), as recent reports on large-area CVD graphene demonstrate mobility values as large as 350,000 (cm^2^ V^−1^ s^−1^) with sheet resistance spanning ~ (*e* ^2^/4*ħ*) to 500(*e* ^2^/4*ħ*) at DC applying only ~ 5 *V*_g_^52^. Our studies reveal the opportunity for a device based on the tunable electronic properties, without complicated fabrication steps required for other THz modulation schemes such as metamaterials^29, 30, 53^, nanophotonic resonators^54^, thin film thickness tuning^37–40, 47^ or materials with switchable metal-to-insulator phase transitions^47–49^.

We have demonstrated that the control of the large-area graphene DC sheet resistance enables the tuning, either electrically or chemically, from the transmission to absorption-dominating regimes at mm wave to THz frequencies. This could have wide-ranging application in the tunable and controllable manipulation of mm wave and THz light, including, for example, gate tunable THz modulators, electronically steerable phased array antennas and radar and tunable variable focus lenses and cones. However, while we have clearly demonstrated a single layer tuned from the transmission to absorption regimes (the gray shaded region in Fig. 4c where *A* > *T*, *R*), we have not pushed well below the inflection point between poor to excellent conductor (the lower bound in Fig. 4c where* R* > *T*, *A*). Future work with increased conductivity (e.g., by improved materials synthesis, using other two-dimensional materials other than graphene, etc) may enable complete tuning from strong transmitter, to strong absorber, to strong reflector, and would enable new technologies to manipulate EM radiation in powerful ways.

## Methods

### CVD graphene growth

Fully continuous monolayer graphene films are grown on copper foils (Alfa Aesar 94555) via chemical vapor deposition (CVD) in a 5-inch-diameter quartz tube. Large-domain (mm-scale grain boundaries) graphene films are synthesized using a fast, oxygen-assisted growth using two stages of methane flow (0.8 and 2.4 sccm flow rates) to separate nucleation and edge growth as presented in ref. ^27^. All gases are left flowing until the furnace cools to less than 300 °C. The entire CVD process occurs with the quartz tube evacuated by a dry scroll pump.

### ODTS SAM deposition

High-resistivity Si wafers with a 90-nm-thick, gate-quality SiO_2_ layer on top are cleaned in hot piranha solution for 1 h followed by cleaning in deionized (DI) water. The wafers are placed in a vacuum chamber with 50 µL of ODTS solution in a glass vial. The chamber is then pumped down and the ODTS is allowed to self-assemble on the SiO_2_ surface for 6 h.

### Graphene transfer

Graphene covering a copper foil (≈ 1 cm^2^) is coated with poly(methyl methacrylate) (PMMA) solution and etched in 5% ammonium persulfate (APS) solution. The floating PMMA/graphene stack is washed in DI water and wet transferred onto the substrate. For BI doping experiments, BI (0.03 M) was dissolved in a solution of APS, H_2_SO_4_ and H_2_O_2_ for copper etching. This process simultaneously etches the copper and dopes the graphene as described in ref. ^31^. Following transfer, the wafer is dried overnight. Before PMMA removal, the wafer is heated on a hotplate at 130 C for 30 min. After cooling to room temperature, the wafer is placed in an acetone bath for 24 h for PMMA removal. The wafers are finally rinsed with isopropyl alcohol, and dried with N_2_ gas.

### Electrical characterization of graphene devices

To probe the electrical properties of the transferred graphene films, four Cr/Au (20/50 nm) electrodes were deposited by electron beam deposition using a shadow mask. All devices use the same shadow mask to maintain a consistent area to be measured. The electrodes were deposited in a square geometry with ≈ 5 mm gap separation between each electrode. The current between two diagonally opposed electrodes, representing the drain and source electrodes, was measured with 100 mV bias applied. Gate voltages were applied through the SiO_2_ layer, with sweeps between −7 and 45 V (experimentally determined window to avoid leakage). Sheet resistance measurements were performed using a lock-in amplifier and a 4-point van der Pauw method.

### THz characterization

An Emcore PB7200 broadband frequency-domain photomixing spectrometer was used to perform THz transmission measurements through the graphene-on-Si etalon structures. As shown in Supplementary Fig. 1, one photomixer acts as a THz transmitter, and a separate photomixer as a THz receiver; one of two distributed feedback lasers is tuned by temperature to perform the broadband difference frequency sweep between 0.2 and 1.2 THz with a 500 MHz step^24^. An aperture was placed between the sample and the THz beam to ensure that the THz beam area (≈ 3 mm diameter) was consistent from sample to sample, and less in area than the graphene film. The THz beam was then aligned at the geometric center of the four electrodes on the graphene film.

### Theoretical calculations

The trend lines according to Eqs. 1 and 2 in Fig. 1b are plotted using a linear variation in *τ*, and *E*_F_ from 50 fs, and 50 meV, to 200 fs and 200 meV (with a step size of 25 fs and 25 meV), respectively. Figure 1c, d plots the conductance value at 100 GHz varying *τ*, and *E*_F_ from 0.5 fs, and 0.5 meV, to 200 fs and 600 meV (with a step size of 0.5 fs and 0.5 meV), respectively.

The transmittance vs. frequency plots for various sheet resistances (dotted lines) in Fig. 3c–h were calculated using the transmission line model as in ref.^40^. The model assumes quasi-plane wave radiation, substrate index of refraction *n* = 3.41 and the graphene sheet is treated as a shunt, lumped impedance, *Z*_g_, where the real part of the graphene impedance (admittance) is equal to the measured DC resistance (conductance), assuming negligible imaginary contributions. The substrate thickness was inputted accordingly during calculation, and variations from device to device are listed in Supplementary Note 12.

The fitting of measured transmittance data follows our previous work as described in ref.^9^. To calculate the AC conductance data points (Fig. 4a, b) the transmission matrix method was performed using a least-mean-squares fit procedure (Supplementary Note 5). For each data point, one transmission peak and two neighboring valleys were used to calculate the admittance (Supplementary Note 13). The thickness of the substrate was varied accordingly for each device (Supplementary Note 12).

The fitting for trend lines in Fig. 4a, b use Eqs. 1 and 2 and a least-means-squares fitting procedure consisting of the real part of the calculated AC conductance values, and the measured DC value to estimate *E*_F_ and *τ* for each device. Up to the final fitting procedure (to estimate *E*_F_ and *τ*), all data fitting consisted of both real and imaginary contributions.

### Data availability

The data that support the findings of this study are available from the corresponding author upon request.

## Electronic supplementary material

Supplementary Information
