# Supplementary material for: Broadband impedance match to two-dimensional materials in the terahertz domain
Source: Nat Commun. 2017 Dec 20;8:2233. doi: 10.1038/s41467-017-02336-z (PMC5738418; doi:10.1038/s41467-017-02336-z)
Supplement: Supplementary file 1 — Supplementary Information [file 41467_2017_2336_MOESM1_ESM.pdf]

Supplementary Information

Broadband THz Spectrometer

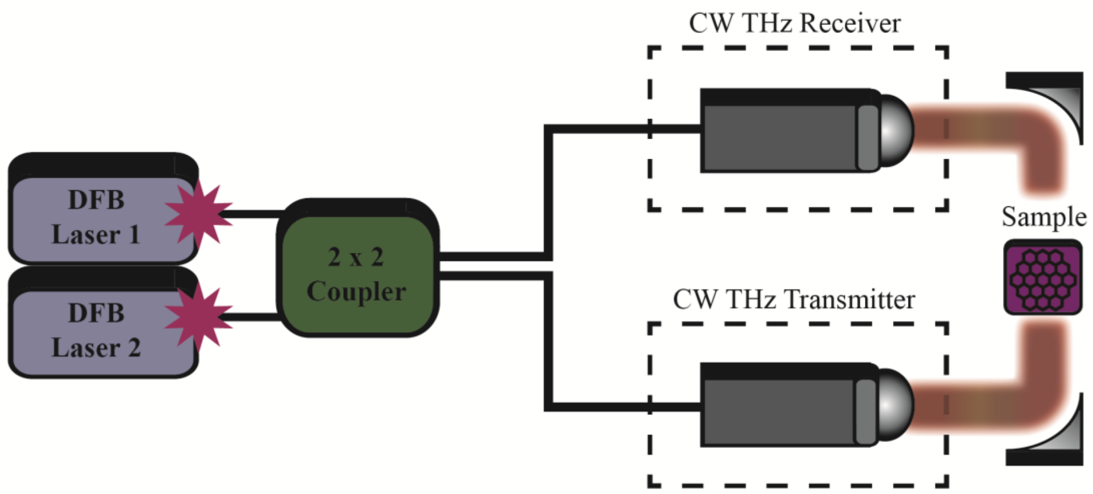

Supplementary Figure 1: Block diagram of the Emcore PB7200 broadband frequency-domain photomixing spectrometer used to perform THz transmission measurements through the graphene-on-Si etalon structures. Two distributed feedback lasers (DFB) are mixed in order to perform broadband frequency sweeps between 0.2 – 1.2 THz with 500 MHz steps.

Supplementary Note 1 - Impedance, Resistance, Conductance

In circuits, impedance means the ratio of the voltage across a given element to the current through a given element, and can be complex (i.e. the current and voltage may be out of phase). An "element" is a physical object with dimensions much smaller than the wavelength at the frequency of interest. The resistance, reactance, admittance, susceptance, and conductance all are various representations of the real or imaginary part of the impedance or its inverse. However, in our experiments, there are no "elements" (all of our structures are of order the wavelength in size), hence one must consider the electromagnetics of waves, not lumped elements.

In electromagnetic waves, the wave impedance means the ratio of the electric field to the magnetic field, and can also be complex (i.e. the electric field and magnetic field may be out of phase). This is also sometimes referred to as the characteristic impedance or the wave impedance of the medium. In cases where the medium is not lossy, the electric field and magnetic field are in phase, and hence the wave impedance is purely real. For example, in vacuum, the wave impedance is 377 Ω. Even though there is no imaginary component, one still generally refers to this as the "characteristic impedance of free space". For all of our experiments, the medium is not absorbing and so the wave impedance is always real. In a medium with index of refraction n (assuming n real as is the case for our experiments), the wave impedance (or also called the characteristic impedance of the medium) is given by 377 Ω/n. Note that a similar definition holds for

the characteristic impedance of a transmission line, which is a distributed inductance and capacitance (and perhaps conductance).

In the context of conducting media such as a metal, the wave impedance has significant imaginary components. In this case, the wave impedance is referred to as the "surface impedance",[1] even for cases where the waves are propagating and there is no physical "surface" in sight. We avoid this definition of "surface impedance" as it does not directly apply to our case.

Instead, we use the concept of sheet resistance. The sheet resistance of a thin film is the ratio of the voltage through a film of width W and length L to the current through that film, divided by the number of squares, i.e. L/W. The concept of sheet impedance is similar; if the voltage and current are out of phase the sheet impedance can have an imaginary component. The sheet conductance is defined similarly. While we consider the case of an imaginary component in the supplementary info for our experiments, we find little evidence for it experimentally in our frequency range and so the main text focuses the sheet resistance only.

The purpose of this paper is to show that, when a plane wave is incident on a thin film, the transmission, reflection, and absorption coefficients of that plane wave depend on the ratio of the characteristic impedance of the wave to the sheet resistance of the thin film.

The quantitative calculation of the transmission, reflection, and absorption coefficients is simplified dramatically using a transmission line equivalent circuit model that captures the key electromagnetic wave phenomena but requires only the use of discreet or disturbed circuit elements, and is described in detail. The model was validated theoretically and experimentally[2].

Supplementary Note 2 - Transmission Line Model

To analyze the THz transmittance of metallic films on dielectric substrates, a transmission-line model can provide accurate values of the transmittance, absorptance, and reflectance over a wide frequency range[2]. The primary requirement is that the incident beam be much wider in extent than the thickness of the substrate + metallic film, and that the beam be at, or near, a "waist". The secondary requirement is that the metallic film be much thinner than a wavelength, which is quite easy to satisfy in the THz region. The "waist" condition means that the constant-phase surface for the beam is a plane perpendicular to the direction of propagation. Hence, because the transmittance depends heavily on the interference effects of propagation in the film + substrate structure, the propagation can be handled as a transverse electromagnetic (TEM) mode. And TEM modes are the basis of the transmission-line model. From a practical standpoint, it is actually more realistic than the standard "plane-wave" model in physical optics because the latter assumes the beam has not only a planar constant-phase surface but also infinite lateral extent - something impossible to achieve in real optical systems.

The equivalent-circuit diagram of the transmission-line model is shown in **Supplementary Fig. 2b**. The incident beam is modelled as a TEM ideal voltage source

having phasor amplitude v_s and source resistance η_0 - the intrinsic impedance of free space. The thin-film-on -substrate structure is represented by the parallel combination of a complex impedance Z_G and a possibly lossy transmission line (to account for substrate absorption effects) of characteristic impedance Z_0. The free space region after the structure is represented by the free-space intrinsic (load) resistance η_0.

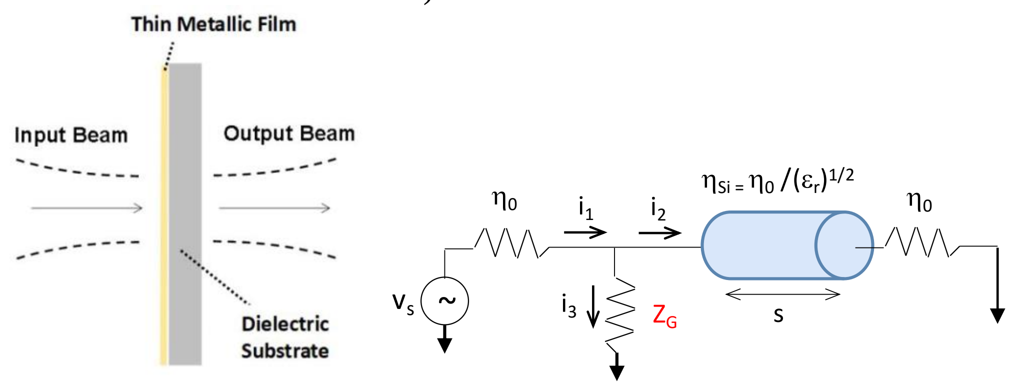

Supplementary Figure 2: a) Beam propagation through a thin metallic film on substrate b) Transmission-line equivalent-circuit model

The combination of transmission line plus free load resistance can be written

$$\eta(s) = \eta_s[\eta_0 + \eta_s \cdot \tanh(\gamma \cdot s)]/[\eta_s + \eta_0 \cdot \tanh(\gamma \cdot s)] \tag{1}$$

where s is the substrate thickness and γ is the complex propagation constant, and η_s is the intrinsic impedance of the substrate = $\eta_0/(\varepsilon_r)^{1/2}$. **Supplementary Eq. (1)** comes from the basic theory of lossy TEM transmission lines such that $\eta(s)$ represents the complex impedance "seen" by a generator connected to the input port[3].

Given the circuit in **Supplementary Fig. 2b**, we can calculate the transmitted power, the power dissipated in the thin-film-on-substrate structure, and the reflected power as follows. We first calculate the phasor currents flowing out of the generator i_1, into the transmission line i_2, and into the thin film i_3, where $i_1 = i_2 + i_3$ by the Kirchoff current law. By voltage division, we then have

$$i_1 = \frac{v_S}{\eta_0 + Z_G \| \eta(s)}, \quad i_2 = i_1 \frac{Z_G}{Z_G + \eta(s)} \text{; and} \quad i_3 = i_1 \frac{\eta(s)}{Z_G + \eta(s)} \tag{2}$$

where the last two follow from current division. Substitution for i_1 then yields

$$i_2 = \frac{v_S}{\eta_0 + Z_G \| \eta(s)} \frac{Z_G}{Z_G + \eta(s)} \tag{3}$$

and,

$$i_3 = \frac{v_S}{\eta_0 + Z_G \| \eta(s)} \frac{\eta(s)}{Z_G + \eta(s)}. \tag{4}$$

The average power dissipated in the thin-film-on-substrate structure is (assuming Ohm's law and sinusoidal phasor)

$$P_2 = \frac{1}{2}|i_2|^2 \operatorname{Re}\{\eta(s)\} = \frac{1}{2}|v_s|^2 \left| \frac{1}{\eta_0 + Z_G \,||\, \eta(s)} \frac{Z_G}{Z_G + \eta(s)} \right|^2 \cdot \operatorname{Re}\{\eta(s)\} \tag{5}$$

and the transmittance T through the metal film is just P_2 divided by the "available power" from the source, $|v_s|^2/8\eta 0$. Hence, we can write

$$T = \frac{1}{2}|i_2|^2 \operatorname{Re}\{\eta(s)\} = 4\eta_0 \cdot \left| \frac{1}{\eta_0 + Z_G \,||\, \eta(s)} \frac{Z_G}{Z_G + \eta(s)} \right|^2 \cdot \operatorname{Re}\{\eta(s)\} \tag{6}$$

By similar reasoning the average power dissipated in Z_G is

$$P_3 = \frac{1}{2}|i_3|^2 \operatorname{Re}\{Z_G\} = \frac{1}{2}|v_s|^2 \left| \frac{1}{\eta_0 + Z_G \,||\, \eta(s)} \frac{\eta(s)}{Z_G + \eta(s)} \right|^2 \cdot \operatorname{Re}\{Z_G\} \tag{7}$$

Such that the thin film absorptance is

$$A = \frac{1}{2}|i_3|^2 \operatorname{Re}\{Z_G\} = 4\eta_0 \cdot \left| \frac{1}{\eta_0 + Z_G \,||\, \eta(s)} \frac{\eta(s)}{Z_G + \eta(s)} \right|^2 \cdot \operatorname{Re}\{Z_G\} \tag{8}$$

By conservation of energy (and power), the reflectance is just

$$R = 1 - T - A \tag{9}$$

This set of equations can handle a wide variety of materials and conditions which are difficult to calculate by conventional (i.e., plane-wave) propagation techniques.

Special Case#1: No Substrate; Purely Resistive Film

The lack of a substrate is the simplest case since according to **Supplementary Eq. (1)** and the behavior of the tanh function, $\eta(s) \rightarrow \eta(s=0) = \eta_0$. Substitution into (6) then yields,

$$T = 4\eta_0^2 \cdot \left| \frac{1}{\eta_0 + (Z_G\eta_0)/(Z_G + \eta_0)} \frac{Z_G}{Z_G + \eta_0} \right|^2 = 4\eta_0^2 \cdot \left| \frac{Z_G}{\eta_0^2(1 + 2Z_G/\eta_0)} \right|^2 \tag{10}$$

substitution into (8) yields,

$$A = 4\eta_0^3 R_G \cdot \left| \frac{1}{\eta_0^2(1 + 2Z_G/\eta_0)} \right|^2 \tag{11}$$

and substitution into (9) yields

$$R = 1 - 4\eta_0^2(\eta_0 R_G + |Z_G|^2) \left| \frac{1}{\eta_0^2(1 + 2Z_G/\eta_0)} \right|^2 \tag{12}$$

In the special case of a resistive film, $Z_G = R_G$, **Supplementary Eq. (10) - (12)** can be greatly simplified algebraically:

$$T = \frac{4R_G^2/\eta_0^2}{(1 + 2R_G/\eta_0)^2} \tag{13}$$

$$A = \frac{4R_G / \eta_0}{(1 + 2R_G / \eta_0)^2} \tag{14}$$

$$R = \frac{1}{(1 + 2R_G / \eta_0)^2} \tag{15}$$

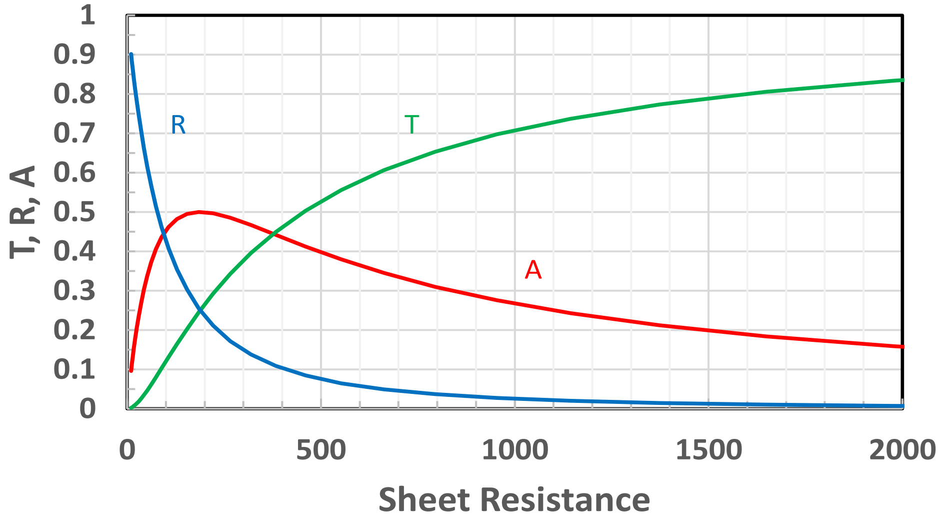

Supplementary Figure 3: The T, R, A values versus sheet resistance for a suspended, purely resistive film.

Interestingly none of these depends on frequency unless R_G is dispersive (more on that later). Historically, **Supplementary Eq. (14)** is known as the Wolsterdorff equation[4].

To check on the physical reasonableness of these last three equations, we note that in the limit of $R_G \rightarrow 0$ (thick metal or "ideal conductor"), T =0, A =0, and R =1 , i.e., we have the "perfect mirror". In the limit, $R_G \rightarrow \infty$ (infinitesimal metal or "ideal insulator"), T =1, A =0, and R =0, i.e., we have the "perfect transmitter". In the special case $R_G \rightarrow \eta_0$, we have T = 4/9, A = 4/9, and R = 1/9. Plots of **Eq. (13) - (15)** vs R_G are shown in **Supplementary Fig. 3**. The value of R_G that maximizes A is $R_G = \eta_0/2$ for which T=1/4, A=1/2, and R=1/4. For all values of R_G, T + A + R = 1, consistent with conservation of power.

Special Case#2: Lossless Substrate; Purely Resistive Film

Because of the difficulty of suspending thin metal films in free space, a more practical case is a substrate of finite thickness but negligible loss. This can be readily achieved even in the THz region by highly-resistive substrates having zero or very low polarity, such as high-resistivity silicon. The low polarity is necessary to make the optical phonons weakly interacting with electromagnetic radiation (silicon having near-zero polarity). In this case, we can re-write **Supplementary Eq. (1)** as

$$\eta(s) = \eta_s [\eta_0 + i\eta_s \cdot \tan(\gamma \cdot s)]/[\eta_s + i\eta_0 \cdot \tan(\gamma \cdot s)] \tag{16}$$

which is the familiar expression from microwave engineering textbooks. Because $\eta_s \neq \eta_0$, substitution into **Supplementary Eq. (6), (8), (10)** then creates a distinct frequency dependence consistent with the well-known behavior of transmission lines that are not

impedance matched to their load. But there are special cases well known from microwave transformer theory that simplify the analysis once again.

For a "half-wave" transformer, $\gamma \cdot s = m(2\pi/\lambda) \cdot \lambda/2 = m\pi$, where m is any integer (including zero) and λ is the wavelength in the transmission-line medium having dielectric constant ε_r, and index of refraction, $n = (\varepsilon_r)^{1/2}$. This condition makes $\tan(\gamma \cdot s) \rightarrow 0$ for all m, so according to **Supplementary Eq. (16)**, $\eta(s) = \eta_0$. And then we recover the same values of T, A, and R, as given by **Supplementary Eq. (13) - (15)**. For a given s, the half-wave condition is satisfied by a set of periodic frequencies $\nu = m \cdot c/(2ns)$, where m = any positive integer (including zero). For a "quarter-wave" transformer, $\gamma \cdot s = (2\pi/\lambda)*\lambda/4 = \pi$, so that $\tan(\gamma s) \rightarrow \infty$, and $\eta(s) = (\eta_s)^2/\eta_0$.

Supplementary Note 3 - Transmission Line Model for Calculating T, R, A

Using the transmission line model, the effect of different substrate dielectric constant/index of refraction can be easily accounted for, and provides a convenient "device design tool". For our experiments where graphene is mounted on a silicon substrate, $\varepsilon_s \approx 11.66$, and a substrate thickness of L = 400 μm is used for the calculations of T, R, and A. These values are compared to a suspended film, and a graphene film on substrate in **Supplementary Fig. 4**. In order to investigate the frequency dependence, in the figure below, the scattering time is set as, τ = 50 fs, as this value is widely reported in the literature (see table below). On half-wave resonance (~ 110 GHz for L = 400 μm), which occur at even integer multiples of $f = \frac{c}{(4*n*L)}$, or when kL = Nπ, the transmittance, reflectance, absorptance values from the transmission line model reduce to the Woltersdorff[4] values. In contrast, at odd integer multiples $f = \frac{c}{(4*n*L)}$, the device is at quarter-wave resonance (~ 164 GHz for L = 400 μm), and mostly reflecting. The equations for T, R, A are listed below. For device design, in the limit where the substrate index of refraction n = 1, and thickness of L = 0, the frequency dependent Fabry-Perot fringes of T, A, R decrease in amplitudes, and approaches the ideal case of a suspended film where the T, R, A values are generally flat with broadband, 50% absorption, matching the Woltersdorff values.

Half-Wave Resonance at Transmittance Peak

It is important to note that, even though the transmission line model matches the Woltersdorff values at the half-wave resonance values (**Supplementary Fig. 4d,h**), the half-wave resonance condition does not always occur at a transmittance peak maxima. The half-wave resonance condition occurs at a transmittance maxima up to a certain critical sheet resistance value (i.e. when the sheet resistance is large), but because of a phase shift when the graphene film becomes reflecting (i.e. when the sheet resistance value is small), the half-wave resonance condition occurs at the transmittance minima[2]. This phenomenon is obvious in **Supplementary Fig. 4e**; for sheet resistance values greater than 156 Ω/sq, the half-wave resonance condition occurs at the transmittance maxima, but for sheet resistance values less than 156 Ω/sq, the half-wave resonance condition occur at the transmittance minima. In this work, we never reach this critical sheet resistance threshold, and thus, in the manuscript, the half-wave resonance condition is referred to at the transmittance peak maxima. The opposite situation occurs for quarter-

wave resonance, that is, for high sheet resistance the quarter-wave resonance occurs at a transmittance minima, but after the critical sheet resistance is reached, the quarter-wave resonance flips and occurs at the transmittance maxima.

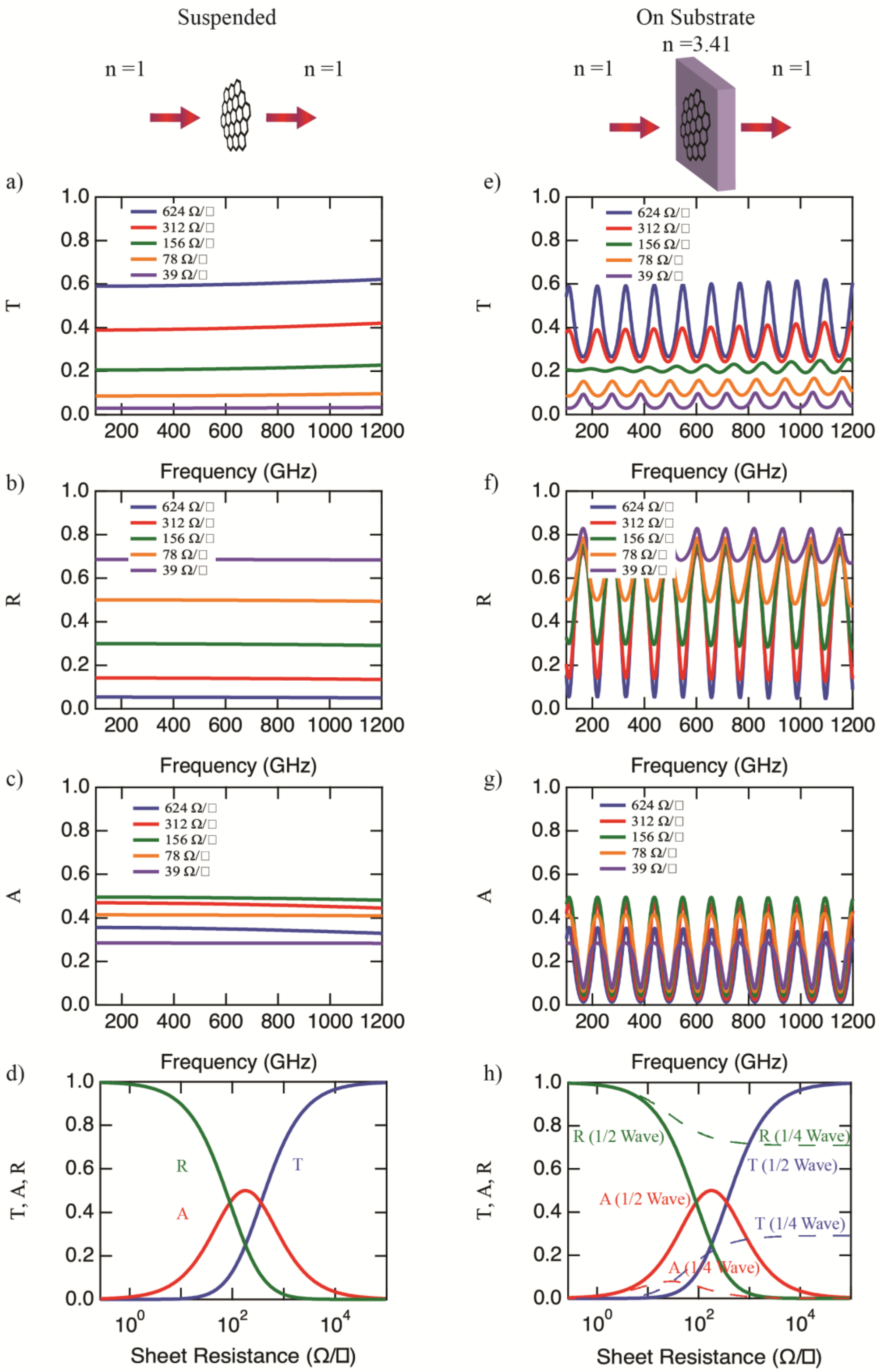

Suspended
n =1
n =1
On Substrate
n =3.41
n =1
n =1
a)
b)
c)
d)
e)
f)
g)
h)
T
R
A
T, A, R
624 Ω/□
312 Ω/□
156 Ω/□
78 Ω/□
39 Ω/□
Frequency (GHz)
Sheet Resistance (Ω/□)
R
T
A
R (1/2 Wave)
R (1/4 Wave)
T (1/2 Wave)
A (1/2 Wave)
T (1/4 Wave)
A (1/4 Wave)

Supplementary Figure 4: Two scenarios were analyzed to investigate the effects of the index of refraction of the substrate used in this work: suspended graphene films, and graphene on a substrate of finite thickness (n = 3.41, L = 400 um) with the THz beam incident on the graphene film first. a-c) The frequency dependent T, A, R was calculated for a suspended film for various assumed values of the DC sheet resistance. d) The single frequency T, A, R values for a suspended film as the sheet resistance is varied. e-g) The frequency dependent T, A, R was calculated for a film on a substrate, using the transmission line model for various assumed values of the DC sheet resistance. At the half-wave resonance, the amplitudes of the transmittance match those of the suspended sheet. h) The half-wave resonance, and quarter-wave resonance values for T, A, R are plotted as the sheet resistance is varied.

Theoretical T, R, A for Suspended Film (Woltersdorff [4])

$$A = \frac{4\,({R_\square}/{Z_0})}{(1+\frac{R_\square}{Z_0/2})^2} \quad (17)$$

$$A = \frac{4\,g}{(1+2g)^2} \quad (20)$$

$$R = \frac{1}{(1+\frac{R_\square}{Z_0/2})^2} \quad (18)$$

$$R = \frac{1}{(1+2g)^2} \quad (21)$$

$$T = \frac{1}{(1+\frac{Z_0/2}{R_\square})^2} \quad (19)$$

$$T = \frac{4g^2}{(1+2g)^2} \quad (22)$$

Quarter-Wave Resonance Case (Incident on Graphene)

$$A = \frac{4\,R_\square Z_0}{|R_\square n + R_\square + Z_0|^2} \quad (23)$$

$$R = \left|1 - \frac{2R_\square}{R_\square n + R_\square + Z_0}\right|^2 \quad (24)$$

$$T = 4{Z_0}^2\left|\frac{R_\square}{2R_\square Z_0 + {Z_0}^2}\right|^2 \quad (25)$$

Supplementary Note 4 - THz Incident on Substrate

The case where the THz beam is incident on the substrate first (with a graphene film on the backside) was investigated analytically with similar results as ref[6]. Contrary to the experimental setup used in this work, that is, where the THz beam is incident on the graphene film, if the THz beam is incident on the substrate side, the transmittance through the etalon remains the same (compared to incidence on the graphene side), but the reflectance and absorptance are different. The absorptance is plotted (with n = 3.41)

varying the graphene sheet resistance and kL varying from 0 – 2π in **Supplementary Fig. 5a** Although the values T, R, A, remain identical at half-wave resonance when kL = Nπ, at quarter-wave resonance, as clearly evident at kL = Mπ, where M is odd integer values, the total reflectance can decrease, resulting in increased absorbance. For this case (which is different from the experimental setup in this work) the absorptance values can be greater than 50% for $n > 1$. The peak absorption found at very low sheet resistance values calculated here were not apparent in similar plots in ref[6] (the sheet resistance where absorption begins to decrease was beyond the range analyzed), but here we emphasize that a sheet resistance dependent maximum absorption does indeed occur. The maximum absorptance, and the sheet resistance value at which it occurs can vary depending on the substrate index of refraction. These values were computed in **Supplementary Fig. 5b**. Here, it is obvious that the largest sheet resistance value for maximum absorbance occurs at 377/2 Ω/sq, when $n = 1$. Absorptance values greater than 90% can be achieved when $n > 3$ with the sheet resistance value required decreasing to values < 100 Ω/sq.

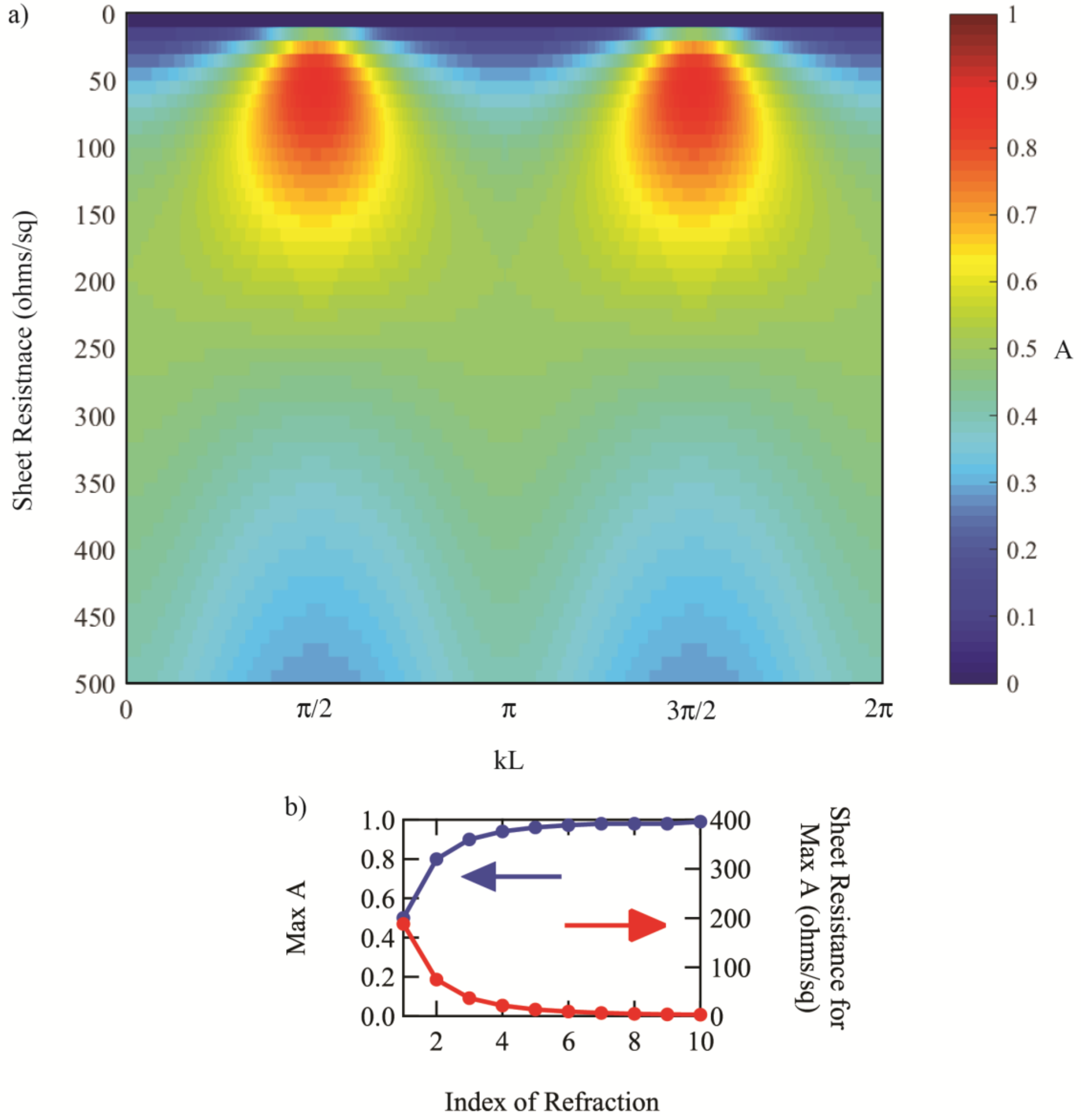

Supplementary Figure 5: a) The absorptance is plotted for the case when the THz beam is incident on the substrate (n = 3.41) side first as a function of the sheet resistance (ohms/sq) with kL varying from 0 – 2π. b) The maximum absorptance, and the sheet resistance required for various values of the index of refraction of the substrate.

T, R, A (Incidence on Substrate Side)

$$T = 4Z_0 \left| \frac{\mathrm{gn}\big(\mathrm{n}\sin(\mathrm{kL}) - \mathrm{i}\cos(\mathrm{kL})\big)}{(1 + \mathrm{g} + \mathrm{gn}^2)Z_0 \sin(\mathrm{kL}) - \mathrm{i}(1 + 2\mathrm{g})\mathrm{n}Z_0 \cos(\mathrm{kL})} \right|^2 \frac{2Z_0}{1 + \mathrm{n}^2 - \cos(2\mathrm{kL})\,(\mathrm{n}^2 - 1)} \qquad (26)$$

$$R = \left|\frac{(1+g-gn^2)\sin(kL)+in\cos(kL)}{(1+g+gn^2)\sin(kL)-i(1+2g)n\cos(kL)}\right|^2 \quad (27)$$

$$A = 4gZ_0^4\left|\frac{n}{Z_0^2((1+2g)n\cos(kL)+i(1+g+gn^2)\sin(kL)}\right|^2 \quad (28)$$

Supplementary Note 5 - Graphene Transfer onto ODTS SAM

CVD grown, monolayer graphene films are transferred onto an ODTS SAM on the SiO_2 substrate depicted in **Supplementary Fig. 6**. The ODTS SAM is vacuum deposited on the wafer prior graphene transfer. During the transfer step, BI can be introduced on the bottom of the film to intentionally dope the graphene. Scanning electron microscopy (SEM) imaging of the transferred graphene film was performed to determine the surface film quality following transfer, shown in **Supplementary Fig. 6b**. Although there are small topological variations, such as bumps and wrinkles created during the transfer process, we observe no detrimental defects (such as holes or tears) in the graphene film. As demonstrated in depletion curves shown in **Supplementary Fig. 7b-c**, graphene films transferred onto ODTS modified substrates exhibit a Dirac-point voltage ~ 22 Vg. This allows greater sensitivity while gating for modulation experiments.

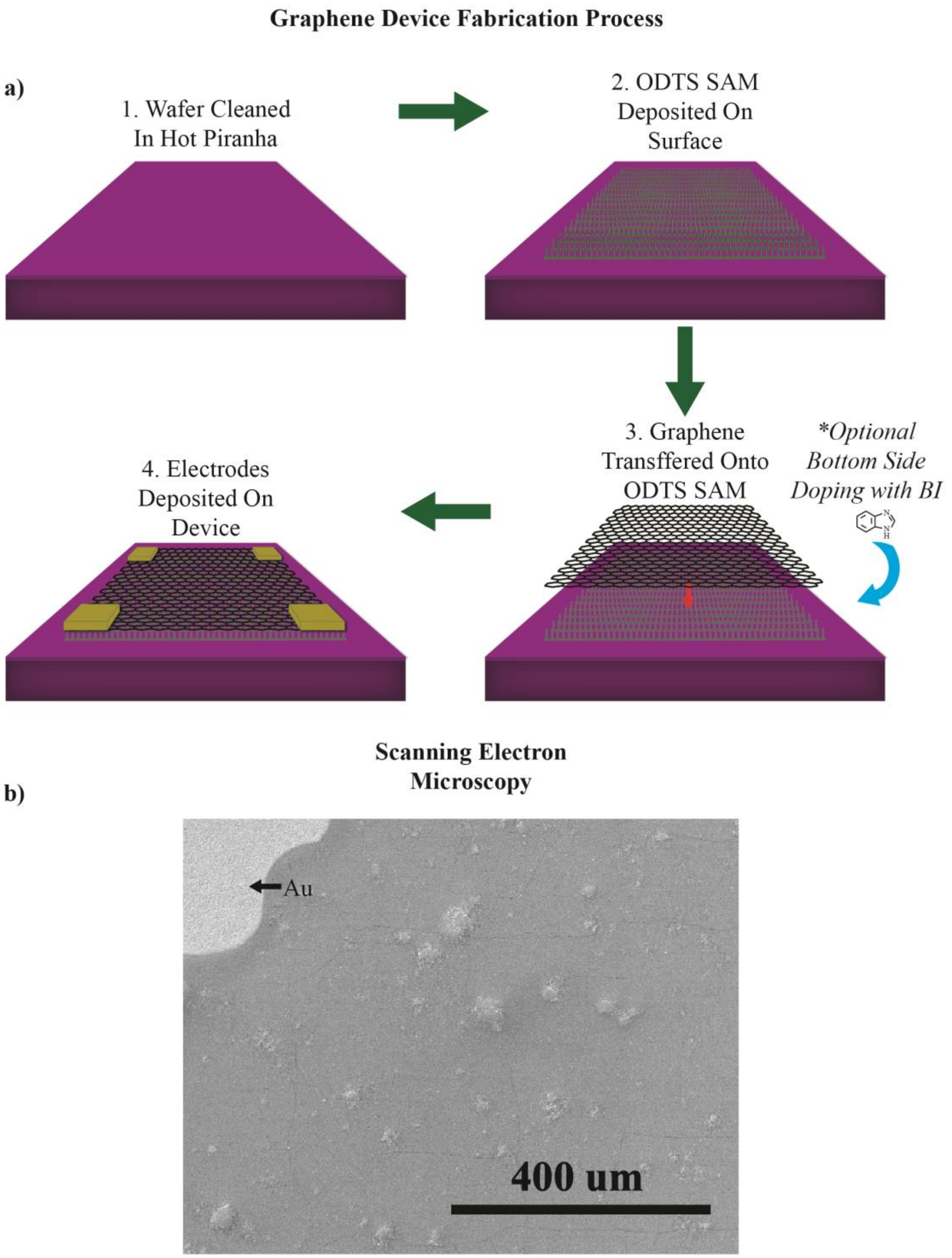

Supplementary Figure 6: a) Diagram of graphene transfer onto ODTS SAM. First a SiO_2 on high-resistivity Si substrate is cleaned in hot piranha solution. This step assists in the vacuum deposition of the ODTS SAM on the substrate surface. Graphene is then transferred using typical wet transfer protocol, with optional BI doping. After the graphene film is transferred and cleaned, electrodes are deposited for electrical measurement. b) SEM image of transferred graphene film. A gold electrode is seen in the top left corner, along with topological bumps and wrinkles from the transfer process.

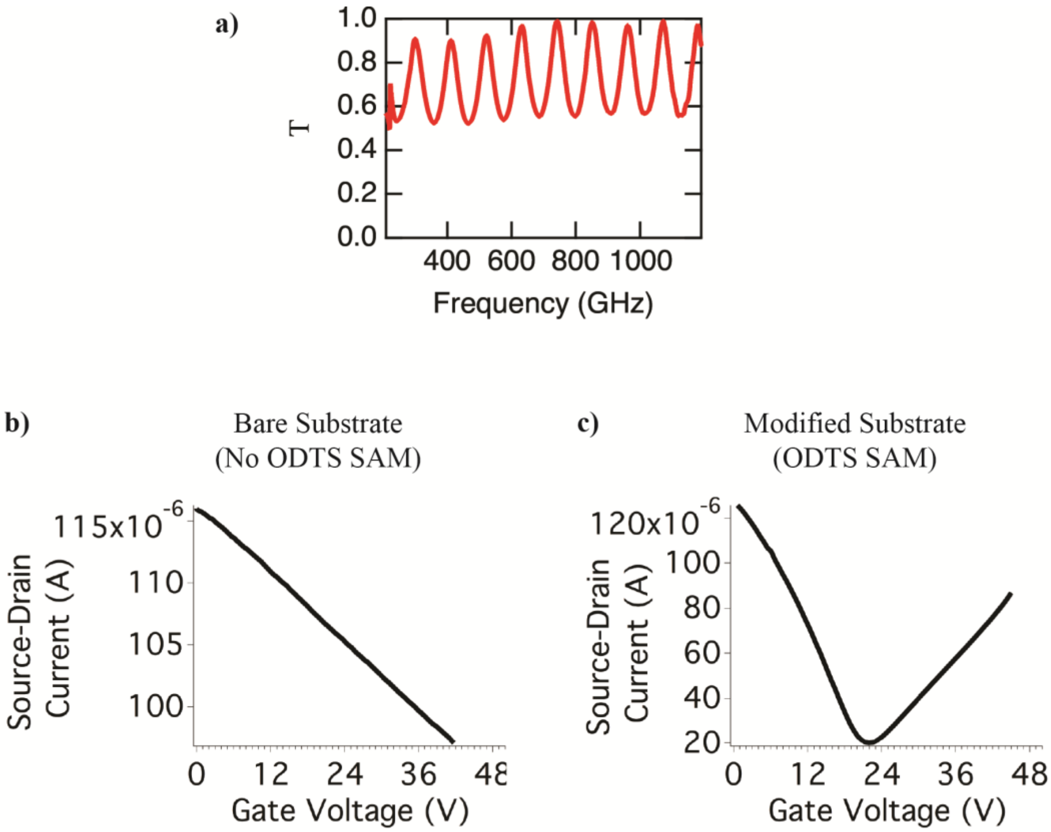

Supplementary Figure 7: a) Transmittance versus frequency of SAM layer on substrate. b) Depletion curve of graphene film on standard substrate without ODTS SAM modification. c) Depletion curve of graphene film on substrate with ODTS SAM modification. The Dirac-point voltage on ODTS SAM modified substrates is ~ 22 Vg, and improves the transconductance.

Supplementary Note 6 - SAM Absorption

Supplementary Fig. 7a shows the transmittance versus frequency of a substrate with a SAM modified surface. The THz transmittance (half-wave resonance) nearly reaches unity across the measured frequency range. In the most extreme circumstance, the transmittance is not completely lossless, but is nevertheless over 90%. This value corresponds to a sheet resistance value of ~ 3500 Ω/sq. Because the SAM resistance is in parallel with the graphene film, the large resistance of the SAM has limited effect when the graphene sheet resistance is low, and results in a difference of less than 15%. The influence of the SAM plays a more significant role when the graphene sheet resistance is comparable (such as when the graphene film is gated to ~ 2000 Ω/sq in **Fig. 3f**). This small absorption contribution from the SAM may potentially explain why the data point (d) deviates from the theory line in **Fig. 4c.**

Supplementary Note 7 - Electrical Modulation of Transmission

The broadband transmittance was measured for a single device at various gate voltages to test the performance as a THz modulator. The peak transmittance (centered at ~ 694 GHz) versus gate voltage is plotted on the same chart as the device resistance, shown below. On a different device (best), the depth of modulation (using +20 V_g and –7 V_g) versus frequency is displayed, and shows frequency variation of the DoM, with a

maximum value of ~ 52%. Variations may arise from inhomogeneous gating using the high resistivity silicon substrate. The use of a high resistivity substrate limits the device switching speed. Experimentally, we measured a modulation frequency of < 2 Hz.

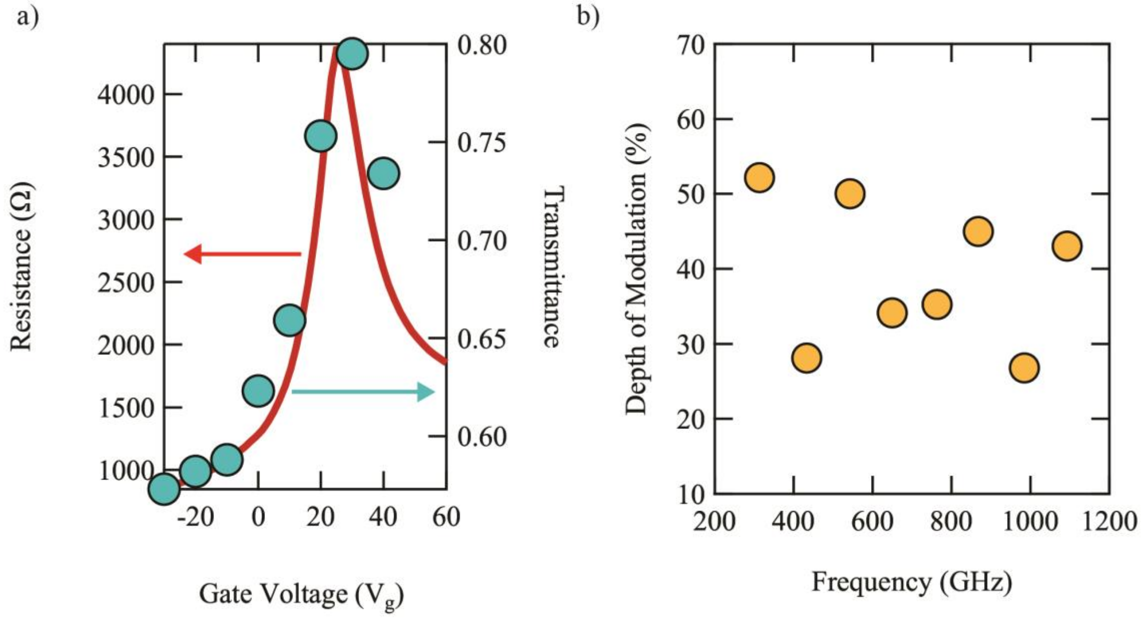

Supplementary Figure 8: a) The resistance versus gate voltage is plotted with the voltage dependent peak transmittance value (located at ~ 694 GHz). b) The depth of modulation of the transmittance peaks for a device under -7 and 20 Vg gate biasing. The maximum value (~ 52%) occurs around ~ 320 GHz, although significant modulation is seen across the broadband frequency range measured.

T_{max} is the maximum transmittance value at a peak of the spectra. Then the transmission variation is given by,

$$\frac{dT_{\max}}{T_{\max}} = -2Z_0\sigma_g \frac{1}{\varepsilon_s + \sigma_g Z_0 + 1} \frac{d\sigma_g}{\sigma_g} \tag{29}$$

where Z_0=377 Ω is the impedance of free space, ε_s is the dielectric constant of the Si substrate and σ_g is the conductance of graphene film. Assuming the device is mounted on a silicon substrate ($\varepsilon_S \approx 11.66$), the room temperature values from Banzerus et. al[7], (mobility ~ 145,000 cm^2/Vs) approaches 100% depth of modulation with only ~10 V on the gate.

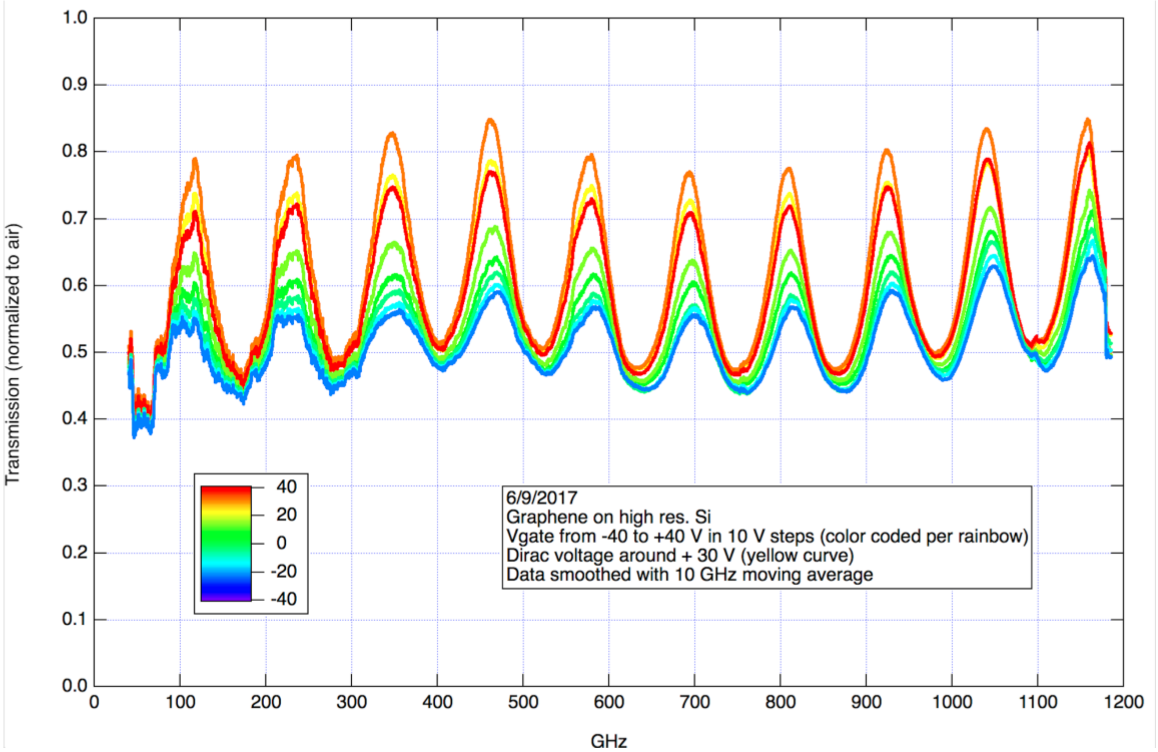

Supplementary Figure 9: Data independently confirming the broadband transmittance amplitude modulating as the graphene sheet resistance is varied (here, by applying a gate voltage on the Si layer) in a different lab using a Toptica Terascan frequency-domain THz spectrometer.

Supplementary Note 8 - Characterizing Doping of Graphene Films

We measured the depletion curve to investigate the effects of doping on the electrical properties of the graphene films. **Supplementary Fig. 10** shows the depletion curves of samples with and without BI doping on an ODTS substrate. After doping, the Dirac point voltage is subsequently shifted to > 48 Vg, indicative that the graphene film becomes hole doped. We found that with the use of BI doping, we could achieve a (zero gate biased) DC sheet resistance below the impedance of free-space. Even with BI doping, the sheet resistance values would vary from sample to sample. The sheet resistance differences from sample to sample could be explained by spatial variations in τ and E_F after doping.

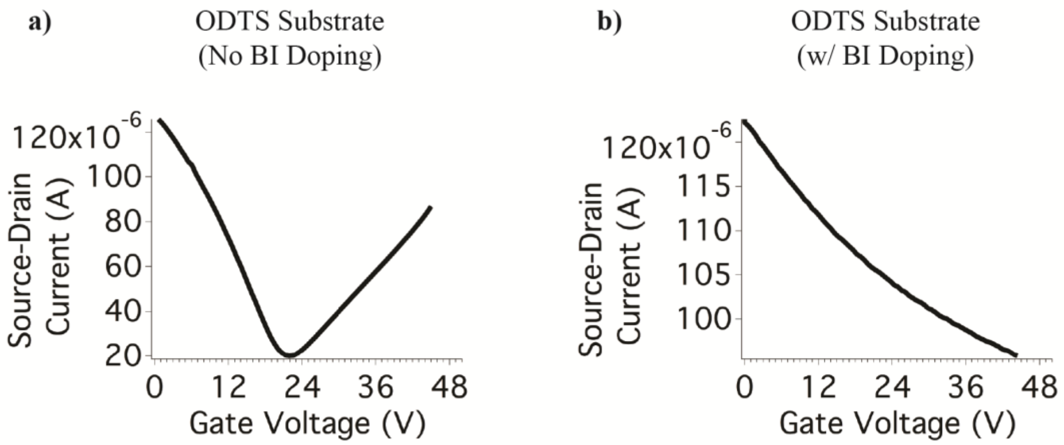

Supplementary Figure 10: Depletion curve data for two samples, with and without BI doping on ODTS substrates. Following doping with BI, the Dirac-point voltage becomes > 48 Vg.

To further investigate the effects of doping graphene, we employed Raman spectroscopy. Raman spectroscopy was performed using a Renishaw Invia Spectrometer with a 532 nm laser. Raman mapping was performed using the Streamline High Resolution (HR) mapping function over a 3 x 3 mm area with 60 um step size. The peak analysis and fitting was performed using the Wire 3.6 Renishaw software package. Mapping images are processed using Igor 6 plotting software.

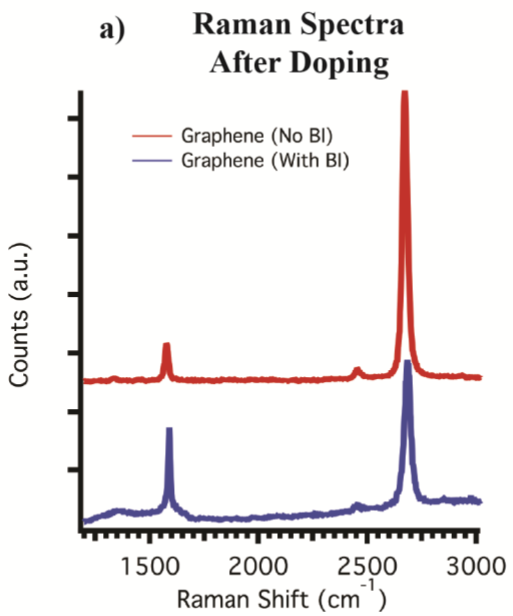

Supplementary Figure 11: Representative Raman spectra for samples with and without BI doping on an ODTS SAM modified substrate. Before doping, the [I]2D/[I]G ratio is much larger than after doping. Also the peak position of the g-peak becomes blue-shifted after doping.

Supplementary Fig. 11 shows representative Raman spectra of graphene samples with and without BI doping during transfer. Notably, after the addition of BI doping, we observe a decrease in the intensity ratio, [I]2D/[I]G. The [I]2D/[I]G ratio is known to

decrease when both the scattering and Fermi energy of the graphene film increase in magnitude[8–10], and thus provides a convenient parameter to assess the BI doping of graphene films. **Supplementary Fig. 12** shows the Raman mapping histograms and images of the [I]2D/[I]G with and without BI doping. We confirm the general trend (lower average in histrogram) of decreased [I]2D/[I]G ratio, but we also observe spatial variance of the graphene film after doping. This supports our belief that after doping, the graphene sheet resistance is not dominated merely by changes in E_F, but instead, by a combination of both E_F and τ. The extracted AC conductivity parameters (outlined below) further support this hypothesis, since under chemical doping, the lowest sheet resistance devices are not the largest E_F, but instead, a combination of moderate E_F and long τ.

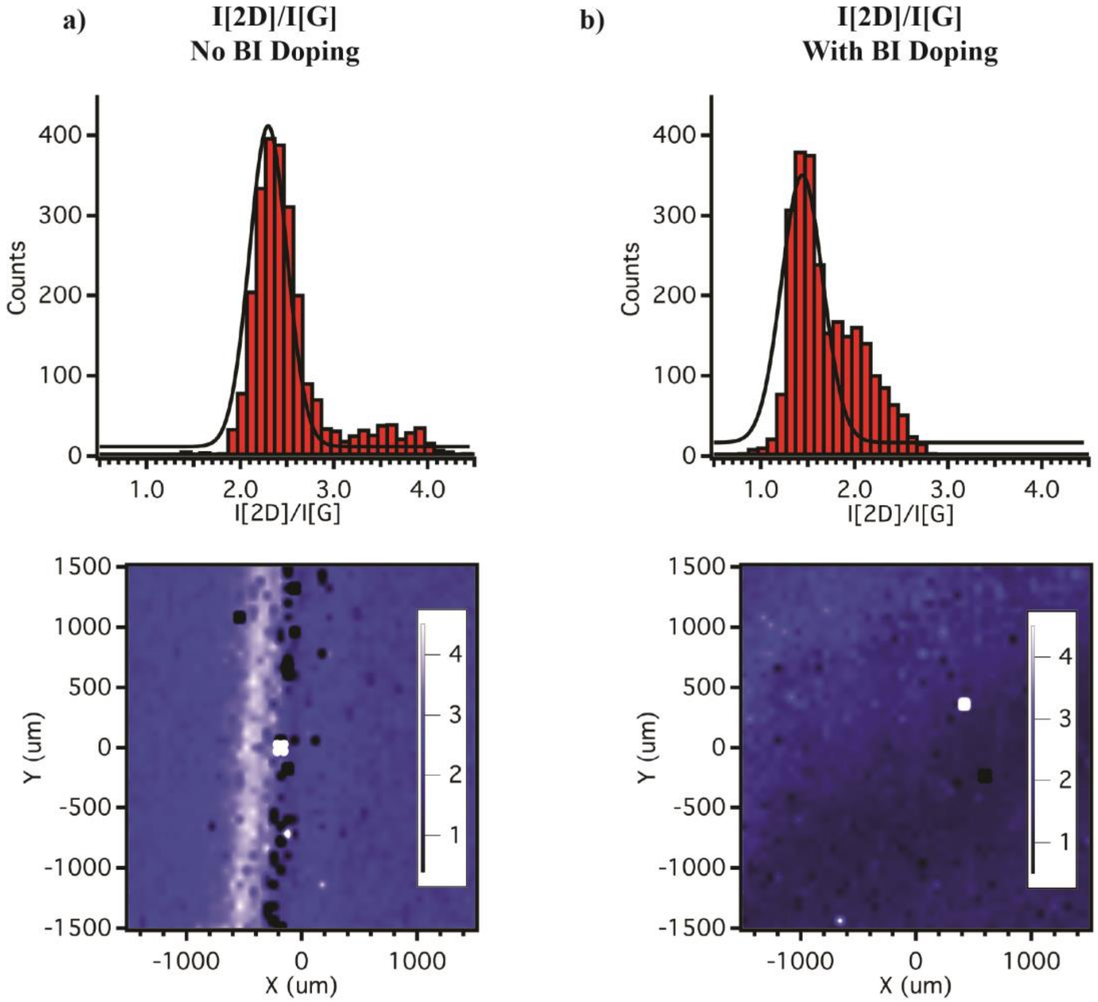

Supplementary Figure 12: Histograms and spatial mapping of [I]2D/[I]G ratio for two samples, with and without BI doping. After doping, the [I]2D/[I]G ratio decreases across the sample.

Supplementary Note 9 - Transmission Matrix Method for Calculating AC Conductance

The transmission matrix method is used[5] for calculating the conductance of graphene films from the transmittance measurement on a substrate of index of refraction, n, and

thickness, L. Assuming the THz beam is normally incident on the graphene surface, the transmittance, T = $|S_{21}|^2$

$$S_{21} = \frac{t_1 t_2 \exp(-ik_s L)}{1 + r_1 r_2 exp(-2ik_s L)} \tag{30}$$

where k_s is the wave vector inside the substrate, and is equal to $k_s = 2\pi\sqrt{\varepsilon_s}/\lambda$. ε_s is the dielectric constant of the substrate, silicon, and λ, is the free-space wavelength. For devices on a silicon substrate, n = $\sqrt{\varepsilon_s}$, with $\varepsilon_s \approx 11.66$ in the THz frequencies. r_1, and t_1, are the coefficients of reflectance and transmittance at the air-Si interface, while r_2, and t_2, are the coefficients of reflectance and transmittance at the Si-graphene/graphene-air interface. These are given by,

$$r_1 = \frac{1 - \sqrt{\varepsilon}_s}{1 + \sqrt{\varepsilon}_s} \tag{31}$$

$$t_1 = \frac{2}{1 + \sqrt{\varepsilon}_s} \tag{32}$$

$$r_2 = \frac{\sqrt{\varepsilon}_s - (\sqrt{\varepsilon}_g + 1)}{\sqrt{\varepsilon}_s + (\sqrt{\varepsilon}_g + 1)} \tag{33}$$

$$t_2 = \frac{2\sqrt{\varepsilon}_s}{\sqrt{\varepsilon}_s + (\sqrt{\varepsilon}_g + 1)} \tag{34}$$

The graphene dielectric constant is a complex value, $\varepsilon_g = Re\{\varepsilon_g\} + iIm\{\varepsilon_g\}$. The complex sheet admittance is computed by,

$$\sigma_g = \frac{\sqrt{\varepsilon_g}}{Z_0} \tag{35}$$

where Z_0 = 377 Ω is the free-space impedance.

Each transmittance peak (consisting of a maximum, and two minimum values) is fitted to calculate the real and imaginary admittance value using a least squares fit procedure. During fitting, the substrate thickness, L, is adjusted for each device being analyzed.

Supplementary Note 10 - Comparison Plot

We now discuss the relationship of this work to prior work on graphene in the THz domain. **Supplementary Fig. 13** shows the range of conductance versus frequency for other monolayer graphene devices previously measured in the literature. Each trend line is plotted using equations (1) and (2), using data points supplied from each reference. The labeled and colored hashed (solid) regions (lines) indicate frequency domain

measurements, whereas the transparent grey regions (lines) are for time-domain systems. The frequency range measured for this paper is one of the broadest (with 500 MHz spectral resolution) investigating graphene-THz coupling, complemented by an extensive range of conductance values, including the achievement of surpassing the free-space impedance threshold.

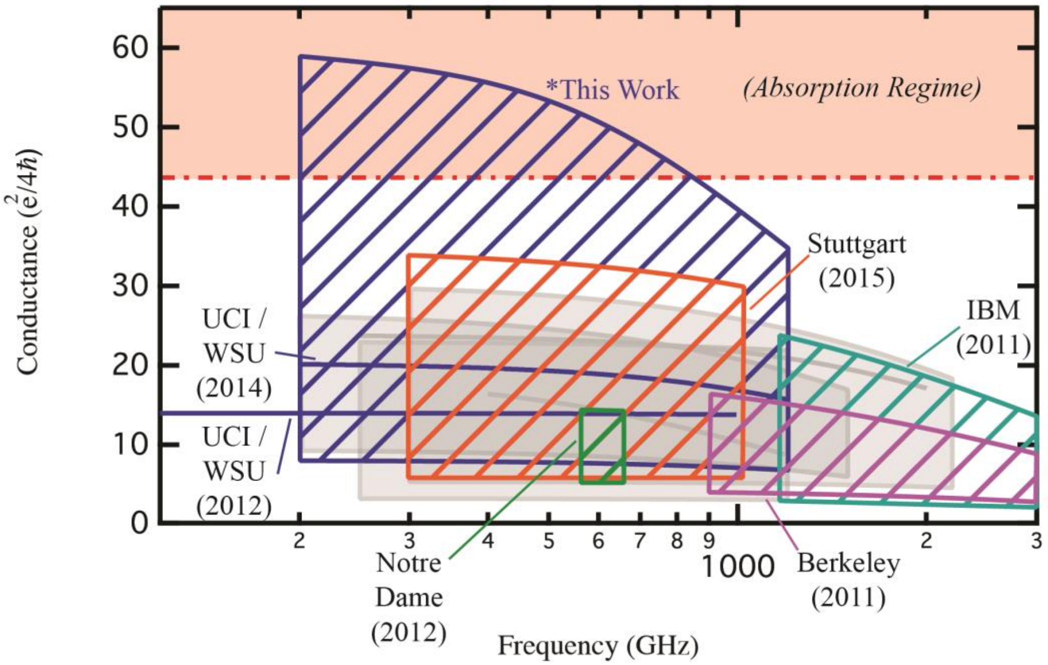

Supplementary Figure 13: Conductance versus frequency range of other graphene devices in the literature. Hashed regions indicate frequency domain systems, while the transparent grey regions show time-domain measurements.

The table below shows both the conductance range and the frequency range of the references used in **Supplementary Fig. 13**. The optical conductance values are those measured and reported from each reference, and τ and E_F are estimated to reflect this value. In reference 8, τ is defined as τ = ħ/Γ, where τ is the scattering time, ħ is the Planck constant, and Γ is the phenomenological scattering parameter, whereas in our calculations, τ is defined as, τ = ħ/2Γ.

Extracted Drude Parameters

Device Measured	DC Sheet Resistance	Modulation Type	τ (fs)	E_F (meV)
Fig. 3d	~ 2000 Ω/sq	Electrical (+20 V_g)	44	105
Fig. 3e	~ 515 Ω/sq	Electrical (-7 V_g)	99	209
Fig. 3f	~ 515 Ω/sq	Chemical Doping	28	583
Fig. 3g	~ 373 Ω/sq	Chemical Doping	74	339
Fig. 3h	~ 250 Ω/sq	Chemical Doping	111	284

Supplementary Table 1: Table of DC resistance, and the extracted E_F and τ values for both electrical and chemical modulation of devices measured in the main text.

Main Text Reference	Frequency Range (GHz)	Measurement Type	Optical Conductance Range (e^2/4hbar)	Estimated to Equal Optical Conductivity	
				τ (fs)	E_F (meV)
Yan et al. #6	1190 -	Frequency Domain (FTIR)	23.7	*23	560
Horng et al. #7	900 -	Frequency Domain (FTIR)	1.9 – 16.09	55	170
Rouhi et al. #8	100 - 1000	Frequency Domain	14.07	50	150
Zhang et al. #9	200 -1200	Frequency Domain	20.63	72	150
Sensale – Rodriguez et al. #10	560 - 660	Frequency Domain	3.28 - 14.8	50	153
Ren et al. #12	100 - 2200	Time Domain	5 - 27.41	50	252
Cervertti et al. #13	300 - 1000	Frequency Domain	3.94 - 36.18	123	157
Min Woo et al. #14	250 - 2100	Time Domain	23.87	50	254
Maeng et al. #15	200 - 1500	Time Domain	5 - 27.09	48.8	295
Buron et al. #16	250 - 1200	Time Domain	3.2 - 23.02	50	246
Mics et al. #18	400 - 1200	Time Domain	19.73	140	70

Supplementary Table 2: Table of frequency range, measurement domain, and achieved conductance range for each reference used in Supplemental Fig. 13.

Supplementary Note 11 - Maximum Absorptance (Incidence on Graphene Side)

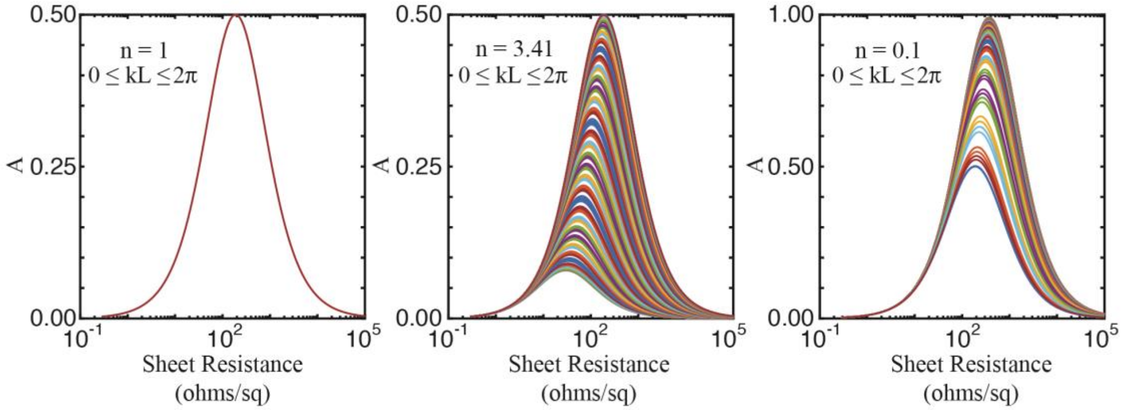

Supplementary Figure 14: The absorption versus sheet resistance for 3 different values of n, while varying kL from 0 to 2π.

The absorptance versus sheet resistance is plotted using the general absorptance **Eq. (7) – (9)** in the case where the THz beam is incident on the graphene side for three values of the n (substrate index of refraction), while varying kL from 0 to 2π.When n = 1 the maximum absorptance is 50%. The maximum absorptance value is also 50% with when n = 3.41 (substrate used in this work) at the half-wave resonance values (kL = Nπ), with a minimum value of ~ 8% in the quarter-wave resonance case. Although **Eq. (7) – (9)** can yield absorptance values greater than 50%, this is only satisfied for values of n < 1, which is not considered in this manuscript, but nonetheless useful for cases such as metamaterials. Hence, 50% is considered the maximum for incidence on the graphene side.

THz Mobility

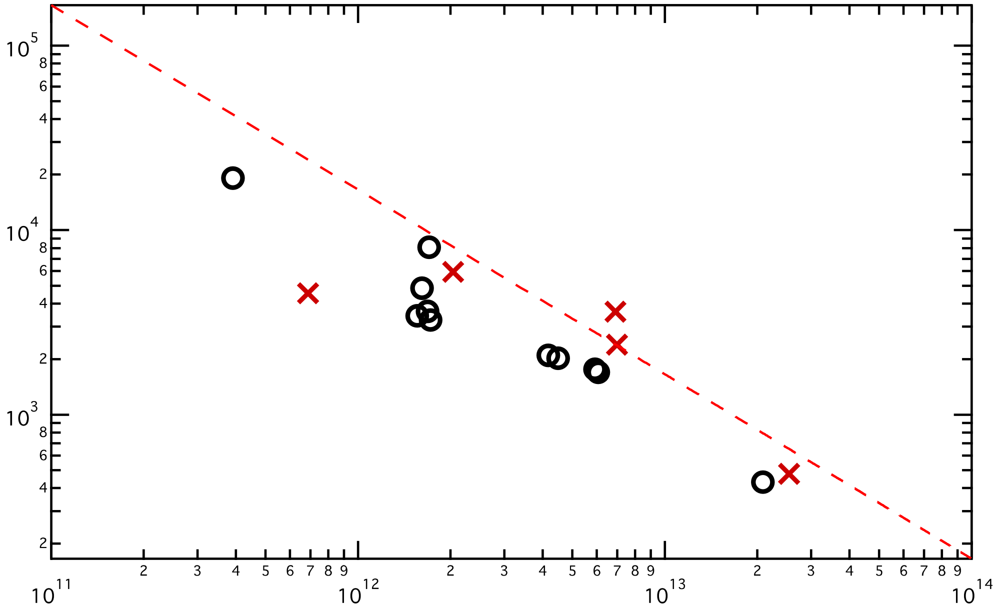

Supplementary Figure 15: The mobility versus carrier density calculated[11] using the scattering time and optical conductivity values from this work, and those reported in Supplementary Table 2. The dotted line shows the 377 Ω threshold.

Supplementary Note 12 - Substrate Thickness

Device Measured	Fig. 3c	Fig. 3d	Fig. 3e	Fig. 3f	Fig. 3g	Fig. 3h
Thickness	395 μm	395 μm	395 μm	398 μm	382 μm	401 μm
Peak-to-peak Spacing	113.48 ± 1.51 GHz	112.04 ± 1.15 GHz	113.23 ± 3.55 GHz	110.74 ± 1.32 GHz	115.50 ± 2.88 GHz	109.64 ± 4.97 GHz

Supplementary Table 3: Table of the effective thickness used for calculation for each device. Also listed are the peak-to-peak spacing averages of the experimental data for each device.

The effective thickness of each device is held constant during calculation and fitting using the values listed above. The peak-to peak spacing of each transmittance peak of all devices were analyzed, and the average values are also listed in **Supplementary Table 3**. Small deviations in the peak-to-peak spacing are apparent. This variation could be due to the common issue collimating the THz beam, where, when the device is not absolutely parallel leads to a spread in the effective substrate thickness. Nonetheless, the peak-to peak spacing agrees with the different effective thickness from the various device. Furthermore, the instrument used is a fiber-based frequency domain photomixing spectrometer; insertion of a sample into the THz beam may shift the interference fringes on the THz arm, leading to a slight off-balance with respect to the optical arm. Therefore, some irregular frequency points in the measured transmittance may be contributed by “instrument drift”. To mitigate the experimental uncertainty from both the instrument drift and beam collimation, post experimental data analysis was employed. Here, we performed fitting to every individual interference pattern around every peak which contains up to hundreds of frequency-transmission points. From the extracted conductance at those individual peaks, we obtain E_F and τ with the Drude model, and compare the calculated DC conductance to the measured DC value, as seen in **Fig. 4a-b**. Here, we find that the two values agree with each other, and supports the reliability of our data.

Supplementary Note 13 - Imaginary Contributions

A Drude-like roll off is expected for frequencies greater than $1/\tau$, as the imaginary contributions become significant, especially when τ is long. During fitting of the measured transmittance peaks, both the real and imaginary contributions were calculated. However, when fitting for τ and E_F only the real contribution is used. Because the Kramers-Koenig relation relates the real and imaginary contributions, a fit on only the real yields valid results[5]. **Supplementary Fig. 16** shows the real and imaginary values calculated, and the calculated Drude trend from the (real part) fitted E_F and τ values for the best device of 250 Ω/sq. The predicted line generally agrees the measured conductance trend. Though the error of the imaginary fit is larger than that on the real part, this error can potentially be improved from a direct measurement of the phase. This would improve the fitting of τ, and would provide a more accurate range of the Drude-shaped roll off $(\omega \sim 1/\tau)$. For our best device (~ 250 Ω/sq), the high frequency

transmittance peaks start to deviate from the predicted trend (when using the measured DC sheet resistance as the real part, ignoring imaginary contributions), suggesting significant contributions from the imaginary. The frequency dependent amplitude deviation is generally not observed in other devices.

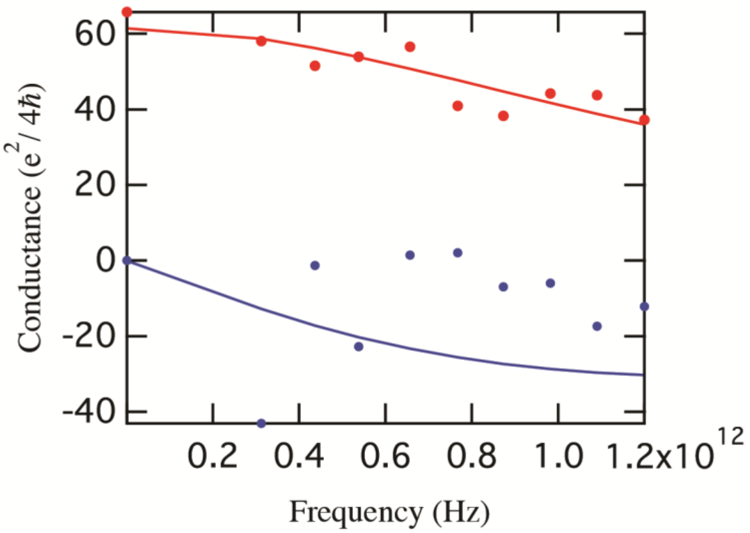

Supplementary Figure 16: The real (red) and imaginary (blue) contributions of the AC admittance calculated by fitting the broadband transmittance data for the best device of 250 Ω/sq. Using the real values, the Drude parameters were calculated, and the Drude prediction is plotted as solid lines.

Supplementary References

1. Dressel, M. & Gruner, G. *Electrodynamics of Solids: Optical Properties of Electrons in Matter*. (Cambridge university press, 2002).
2. Brown, E. R., Zhang, W. D., Chen, H. & Mearini, G. T. THz behavior of indium-tin-oxide films on p-Si substrates. *Appl. Phys. Lett.* **107,** 91102 (2015).
3. Pozar, D. M. *Microwave Engineering*. (J. Wiley, 2005).
4. Woltersdorff, W. Uber die optischen Konstanten dunner Metallschichten im langwelligen Ultrarot. *Zeitschrift fur Phys.* **91,** 230–252 (1934).
5. Zhang, W., Pham, P. H. Q., Brown, E. R. & Burke, P. J. AC conductivity parameters of graphene derived from THz etalon transmittance. *Nanoscale* **6,** 13895–9 (2014).
6. Zanotto, S. *et al.* Coherent absorption of light by graphene and other optically conducting surfaces in realistic on-substrate configurations. *APL Photonics* **2,** 16101 (2017).
7. Banszerus, L. *et al.* Ultrahigh-mobility graphene devices from chemical vapor deposition on reusable copper. *Sci. Adv.* **1,** e1500222 (2015).
8. Ferrari, A. C. *et al.* Raman Spectrum of Graphene and Graphene Layers. *Phys. Rev. Lett.* **97,** 187401 (2006).
9. Das, A. *et al.* Monitoring dopants by Raman scattering in an electrochemically top-gated graphene transistor. *Nat. Nanotechnol.* **3,** 210–215 (2008).
10. Lee, W. H. *et al.* Control of Graphene Field-Effect Transistors by Interfacial Hydrophobic Self-Assembled Monolayers. *Adv. Mater.* **23,** 3460–3464 (2011).
11. Tan, Y. W. *et al.* Measurement of scattering rate and minimum conductivity in graphene. *Phys. Rev. Lett.* **99,** 246803 (2007).
